# Materials challenges in solid-state sensors for continuous monitoring of ions in water

**DOI:** 10.3762/bjnano.17.66

**Published:** 2026-07-27

**Authors:** Maryam Darestani-Farahani, Peter Kruse

**Affiliations:** 1 Department of Chemistry and Chemical Biology, McMaster University, 1280 Main Street West, Hamilton, Ontario L8S 4M1, Canada

**Keywords:** aqueous ion sensors, biosensors, chemical sensors, nanomaterials, water quality monitoring

## Abstract

Accurate detection of ions in aqueous environments, ranging from trace to high concentrations, is essential for monitoring natural water resources, treatment facilities, and wastewater systems. Solid-state sensors have emerged as versatile platforms for this purpose due to their adaptability in geometry, compatibility with electronic integration, portability, and low energy requirements. A wide variety of active materials have been investigated, including metal oxides, graphene, carbon nanotubes, silicon nanowires, AlGaN/GaN, MXenes, transition metal dichalcogenides, and organic polymers all of which can be tailored for sensor development. Sensor selectivity and functionality can be enhanced through strategies such as defect engineering, nanoparticle doping, surface functionalization with organic molecules, or incorporation of advanced recognition elements like metal–organic frameworks, covalent organic frameworks, ion-imprinted polymers, and biomaterials. Hybrid nanocomposites and ion-selective membranes further expand the design space, enabling customized performance for specific applications. This review systematically examines the materials employed in solid-state ion sensors, their transduction mechanisms, and analyte interactions with sensing media, while critically evaluating advantages, limitations, fabrication approaches, and performance metrics. In addition, recent advances in sensor arrays are highlighted to demonstrate progress toward multiplexed detection. By consolidating these developments, this work provides a comprehensive framework to guide the rational selection of active and sensing materials for designing electrochemical and electrical devices capable of reliable, real-time ion monitoring in water.

## Introduction

Access to clean water is critical for public health and ecosystem integrity. Traditionally, water quality monitoring has relied on periodic grab sampling and laboratory analyses. While useful, these methods only offer brief snapshots and may miss sudden changes or localized chemical shifts. For example, ion levels in natural, treated, or distributed water can change rapidly, within minutes or hours, due to factors like storm runoff, treatment plant issues, pipe leaks, or ion exchange processes such as those in softening systems. Continuous online monitoring provides significant benefits in these situations. Without frequent monitoring, these fluctuations might go unnoticed until routine checks reveal a problem [1]. Additionally, many regulations such as WHO guideline values, the U.S. EPA National Primary Drinking Water Regulations and European Union set strict limits on ion concentrations, and real-time monitoring allows for early warnings and quick corrective actions [2–5]. In large-scale water systems, integrating widely deployed sensors with telemetry creates intelligent networks that enhance efficiency, identify issues like contamination or corrosion, and inform infrastructure decisions. Ultimately, online monitoring transforms water systems from passive sampling regimes to active management systems [1].

Electronic ion-sensing platforms including electrochemical (ion-selective electrodes (ISEs) [6–7], amperometric and voltammetric sensors) and electrical (field-effect transistors (FETs) [8–10] and chemiresistors [11]) are promising for online deployment due to their portability, low power, and amenability to automation and networking. To be fit for purpose in online monitoring, sensors must meet requirements that extend beyond laboratory figures of merit. (i) Selectivity is essential to discriminate targets in complex matrices containing abundant co-ions and natural organic matter; this requires an understanding of unwanted interferences for the system [8–10] and smart design, modification, and protection of the active surface according to the system. (ii) Sensitivity, limit of detection (LOD), and dynamic range must satisfy regulatory thresholds, for trace metals often in the low-micrograms-per-liter regime; for nutrients, typically milligrams per liter or lower [2–3,12]. (iii) Calibration stability and drift control are central for continuous operation because frequent recalibration is impractical. Sensors must maintain their performance over extended periods (weeks to months or even years) with minimal drift, or include self-calibration or diagnosis capability [7,13–15]. (iv) Response times should be fast to capture transients without excessive averaging. (v) Accuracy in a variable sensing matrix and with temperature fluctuations is required because real samples (biological fluids, environmental solutions, or industrial streams) differ widely in composition and operating conditions. In practice, this means the device must tolerate variations in ionic strength, pH, temperature, and fluid flow since these factors influence ion activity, binding equilibria, charge screening, and the electrical response of the transducer. (vi) Fouling resilience and mechanical stability are also important parameters. In real water systems, sensors are exposed to biofilm growth, particulate loading, scaling, and mechanical stress. Designs must mitigate fouling and maintain performance in spite of these conditions [16]. Finally, (vii) the sensor should be suitable for in situ (on-site) measurements and operate with low power requirements to support long-term use. It should also allow for automatic data recording and wireless data transmission, be housed in a durable and weather-resistant enclosure and operate reagent-free and with little or no routine maintenance. Ideally, the system should also support network connectivity to enable remote access and continuous monitoring [17].

In summary, electronic ion sensors especially ISEs, FETs, and chemiresistive devices, are central to next-generation online water quality monitoring. They offer sensitivity and selectivity aligned with regulatory thresholds, fast response to capture transient events, and the potential for dense spatial networks. Given these advantages, there are recent reviews highlighting rapid advances in all-solid-state ISEs [18], FET devices and electrochemical sensors for ion detection in water [19–21], and integrated systems that couple sensing with communications and analytics for field-deployable monitoring [22–24]. These are not going to be covered in this review. This review aims to critically assess the diverse classes of nanomaterials as active layer for electrodes, FETs, and chemiresistors, and as sensing element for these solid-state electronic sensors; this is to understand how different materials influence performance, stability, and applicability of the devices in continuous water quality monitoring. Here we provide an overview of application and device geometry of electrical sensors employed for online monitoring of ions in aqueous environments, with a focus on recent advancements in the materials utilized in water quality monitoring. Furthermore, the specific challenges associated with each class of sensing materials will be examined, followed by discussion of emerging strategies and innovative approaches to enhance their performance in water quality monitoring.

## Review

### Applications

The desired properties of sensor materials are driven both by the need for selective interaction with the analyte of interest in the relevant concentration range and the need for tolerating the presence of the analyte matrix. We therefore start with a brief overview of relevant areas of application for aqueous ion sensors. The rapid and continuous assessment of ionic species in natural water systems and drinking water, industrial water, wastewater, and swimming pools has become increasingly critical for ensuring water quality, detecting contamination events, and supporting process control in treatment facilities. Among the ions of primary concern, nutrients are widely monitored due to their roles in nutrient cycling, eutrophication, and as indicators of agricultural runoff and wastewater contamination [14,25–26]. Common cations and anions behave like persistent fingerprints that can be used to track source water mixing, intrusion, or salinity variations [4,14,26–27]. Transition metal ions are important for evaluating corrosion, leaching, and industrial contamination in distribution systems and natural waters [28–30]. Continuous monitoring of these ionic species can provide early warnings of pollution events, treatment failures, or changes in source water chemistry, enabling rapid management responses. Recent developments in sensing technologies have made real-time and in situ measurements of these ions increasingly feasible, advancing toward integrated, data-driven water quality management [14,25,31].

#### Nutrients

Continuous monitoring of nitrate, nitrite, and ammonium is increasingly essential in drinking water, domestic wastewater, and industrial wastewater because these nitrogen species are contaminants that are subject to regulatory limits and serve as real-time indicators of treatment performance. In drinking water, nitrate and nitrite are regulated due to methemoglobinemia risks [32], with limits of 50 mg/L nitrate and 3 mg/L nitrite according to the WHO (Table 1) [2]. Ammonium is not universally regulated but is widely monitored because its presence promotes nitrification and indicates contamination or chloramination instability; online sensors typically require detection limits of 0.005–0.05 mg/L for nitrate and nitrite and ≈0.01 mg/L for ammonium to provide early warning in distribution systems (Table 1) [33–34]. In domestic wastewater, ammonium is the primary pollutant removed through nitrification, while nitrate and nitrite serve as operational markers of biological nitrogen removal; effluent limits commonly target 1–5 mg/L NH_4_^+^–N or 10–15 mg/L total nitrogen, necessitating online detection limits of 0.05–0.1 mg/L for nitrite and nitrate and 0.1–0.5 mg/L for ammonium to support aeration and carbon-dosing control [35–37]. In industrial wastewater, nitrogen monitoring is particularly crucial in fertilizer and chemical production, food and beverage processing, aquaculture, mining, metal finishing, and semiconductor manufacturing, where ammonium, nitrite, or nitrate may represent regulated pollutants, corrosive agents, or indicators of process losses. Industrial analyzers therefore combine low detection limits (0.01–0.1 mg/L) with wide analytical ranges extending to hundreds of milligrams per liter, enabling control of both high-strength process streams and low-concentration effluents [38–40].

**Table 1:** A list of nutrients and common cations and anions with their sources and their health and environmental effects together with the WHO guideline maximum value for drinking water.

Ion	Sources	Health and environmental effects	WHO guideline	Ref.

nitrate (NO_3_^−^)	fertilizers, septic leakage, agricultural runoff	high levels cause methemoglobinemia (blue-baby syndrome) in infants	50 mg/L	[2,57]
nitrite (NO_2_^−^)	intermediate in nitrification/denitrification, contaminated pipes	more toxic than nitrate; causes methemoglobinemia and blood oxygen transport issues	3 mg/L	[2,57]
ammonium (NH_4_^+^)	biological decay, sewage, fertilizers	indicates pollution; affects chlorination efficiency; high levels may affect taste/odor	0.5 mg/L (Rec)	[2,57]
sodium (Na^+^)	natural minerals, seawater intrusion, softeners	high sodium intake is of concern for individuals with hypertension	200 mg/L (Rec)	[2,43]
potassium (K^+^)	natural sources, fertilizers, softening processes	essential nutrient; toxic only at extremely high intake; no typical water toxicity	—	[2,43]
calcium (Ca^2+^)	natural minerals (limestone, gypsum)	essential mineral; contributes to water hardness; no known toxicity in water	—	[2,42]
magnesium (Mg^2+^)	natural minerals, seawater intrusion	essential mineral; excessive levels contribute to water hardness and laxative effects	—	[2,42]
fluoride (F^−^)	natural groundwater, industrial emissions	prevents dental caries at low levels; causes fluorosis at high levels	1.5 mg/L	[2]
chloride (Cl^−^)	natural salts, seawater intrusion, industrial waste	not toxic at normal levels; affects taste and corrosion	250 mg/L (Rec)	[2]
bromate (BrO_3_^−^)	formed during ozonation of water with bromide	carcinogenic (kidney tumors observed in animals)	0.01 mg/L	[49]
carbonate/hydrogen carbonate (CO_3_^2−^/HCO_3_^−^)	natural rock weathering; alkalinity	nontoxic; important for buffering capacity and pH stability	—	[51]
sulfate (SO_4_^2−^)	natural minerals, mining, industrial discharge	high levels can cause diarrhea (especially with Mg/Na) and taste effects	500 mg/L (Rec)	[2]
phosphate (PO_4_^3−^)	fertilizers, detergents, wastewater	not typically toxic at drinking-water levels; contributes to eutrophication in surface water	—	[56–57]

#### Common cations

Real-time, continuous monitoring of the major cations, sodium, potassium, magnesium, and calcium, is important because these ions control both salinity and hardness, which in turn affect health-related guidelines, aesthetics, and treatment performance. Regarding drinking water, the EU Drinking Water Directive and the WHO set a recommended value of 200 mg/L for sodium (primarily for taste and for individuals with hypertension, heart disease, or kidney problems). While there are no EU-wide limits for calcium and magnesium, they are managed via hardness and taste thresholds; WHO and related reviews report calcium taste thresholds of roughly 100–300 mg/L (as CaCO_3_) and note that hardnesses above 200–500 mg/L as CaCO_3_ are generally considered poor or unacceptable from an operational and consumer perspective (Table 1). Continuous cation monitoring in drinking water systems therefore benefits from low-milligram-per-liter detection limits (0.5–1 mg/L for Na^+^ and K^+^; 0.1–1 mg/L for Ca^2+^ and Mg^2+^) so that utilities can detect deviations well below these operational thresholds and manage blending, softening, or desalination processes in real time [2,5,41–43]. In domestic wastewater, Na^+^, Ca^2+^, and Mg^2+^ govern bulk salinity and hardness, which strongly influence activated-sludge flocculation, nitrification, and sludge dewatering; experimental studies show that increased NaCl loads can alter flocculation and, at higher levels, inhibit biological treatment, while hardness (mostly Ca^2+^ and Mg^2+^) can improve sludge flocculation strength and dewaterability. Here, online cation sensors with milligram-per-liter-level detection limits, with ranges extending to several grams per liter, are appropriate to follow storm-driven dilution and episodic saline shocks [44–45]. In industrial wastewater, continuous monitoring of these common cations is especially critical in high-salinity sectors, such as textile dyeing, tanning, petroleum and petrochemical processing, food and beverage production, desalination brine management, and coastal plants impacted by seawater intrusion, where Na^+^, Ca^2+^, and Mg^2+^ dominate salinity, affect membrane fouling, drive corrosion and scaling, and can inhibit biological nitrogen removal at elevated concentrations. In these applications, real-time cation measurements with broad dynamic ranges (from few milligrams per liter up to several tens of grams per liter as total dissolved salts) provide the resolution needed for proactive process control, early detection of saline upsets, and demonstration of compliance with site-specific salinity and hardness criteria [46–48].

#### Common anions

Common anions, including fluoride, chloride, bromate, sulfate, carbonate/hydrogencarbonate, and phosphate, strongly influence corrosivity, scaling, eutrophication risk, disinfection by-product formation, salinity, and regulatory compliance. In drinking water, several anions have strict health-based or operational limits under the EU Drinking Water Directive and WHO guidelines, including fluoride (1.5 mg/L) and chloride (250 mg/L), while bromate, a carcinogenic disinfection by-product, has a stringent maximum of 10 µg/L, requiring sub-microgram-per-liter detection for early warning of ozone or advanced-oxidation-process upsets [2,5,49]. Continuous monitoring of alkalinity species (carbonate/hydrogencarbonate, phosphate) is also essential for controlling corrosion, buffering capacity, and softening processes (Table 1) [2,50–53]. In domestic wastewater, anions such as sulfate and chloride are key drivers of salinity that can inhibit nitrification at elevated levels [54–55], while phosphate is a regulated nutrient contributing to eutrophication; typical effluent limits in Europe and international guidelines require total phosphorus levels of ≤1–2 mg/L, making online orthophosphate monitoring with sub-milligram-per-liter detection limits vital [37,50,56–59]. In industrial wastewater, anion monitoring is especially critical in semiconductor manufacturing (fluoride, chloride, sulfate) [60–61], pulp and paper (chloride, sulfate) [62], textile dyeing (chloride, sulfate) [63], petrochemical and refinery operations (chloride, sulfate) [64], fertilizer and food industries (phosphate), and desalination plants (bromide/bromate and chloride) [5,65]. These sectors face both regulatory discharge requirements and process-control challenges, often necessitating continuous analyzers with detection ranges from low microgram per liter (e.g., bromate) to thousands of milligrams per liter (e.g., chloride or sulfate in industrial brines) [5]. Thus, real-time anion monitoring provides essential high-resolution data for maintaining regulatory compliance, protecting infrastructure, optimizing treatment processes, and mitigating environmental impacts across all water sectors.

#### Heavy metals

Lead, mercury, arsenic, chromium, cadmium, manganese, iron, nickel, and copper are important heavy metals. Lead, iron, and copper may enter drinking water due to the corrosion of plumbing materials in the distribution system. Mercury can also appear in the form of methylmercury, the most toxic and environmentally prevalent species, alongside its ionic form. Arsenic can naturally occur in groundwater and unlike most of metals, which are typically present as cations, arsenic commonly exists as oxoanions (arsenite or arsenate). Other metals occur as oxoanions only in their higher oxidation states, notably chromium(VI) and manganese(VII), which form chromate (or dichromate) and permanganate [4,42,66–67].

Because toxicity, mobility, and regulatory relevance of many metals depend strongly on their oxidation state, it is essential to distinguish these species explicitly in both discussion and tabulated summaries. For example, Cr^6+^ is a carcinogenic ion with high mobility and strong oxidizing character, whereas Cr^3+^ is far less toxic, less mobile, and even an essential micronutrient at trace levels. A similar contrast exists for arsenic, where As^3+^ is more toxic, more mobile, and more weakly adsorbed, while As^5+^ is comparatively less toxic and more strongly retained by mineral surfaces. Iron and manganese also exhibit oxidation-state-dependent behavior: Fe^2+^ and Mn^2+^ are soluble under reducing conditions, whereas Fe^3+^ and Mn^4+^ readily precipitate as oxides, influencing both their detectability and their appearance or operational impacts in water systems. These distinctions are critical for sensor design because the target oxidation state determines the required detection chemistry and redox environment.

Their online monitoring is essential in drinking water, mining effluents, and domestic and industrial wastewater due to their toxicity and stringent regulatory limits. In drinking water, the EU Drinking Water Directive imposes very low maximum acceptable concentrations (MACs) such as for lead (10 µg/L), arsenic (10 µg/L), mercury (1 µg/L), chromium (50 µg/L), nickel (20 µg/L), manganese (50 µg/L), and copper (2 mg/L), necessitating online or high-frequency monitoring solutions with low-microgram-per-liter detection limits for early detection of contamination events, corrosion-release episodes, and treatment failures [5]. In domestic wastewater, heavy metals entering from sewer networks and industrial contributions can inhibit biological nutrient removal, impair sludge quality, and cause biosolids to fail land-application standards; although municipal effluent limits vary by Member State, typical discharge permits restrict metals to ranges of 1–100 µg/L for highly toxic species (Cd^2+^, Hg^2+^, Pb^2+^, As^3+^/As^5+^) and 0.1–2 mg/L for Fe^2+^/Fe^3+^, Mn^2^/Mn^4+^, Ni^2+^, Cu^2+^, or Cr^6+^/Cr^3+^ ions, requiring sensors capable of tracking both trace concentrations and episodic spikes [68–70]. In industrial wastewater, continuous heavy-metal monitoring is particularly critical in electroplating, battery manufacturing, semiconductor production, mining/metallurgy, chemical synthesis, pulp and paper, fertilizer production, petroleum refining, and textile dyeing, where metal concentrations can fluctuate rapidly and effluent limits are often extremely stringent (frequently <10–50 µg/L for Cd^2+^, Hg^2+^, Pb^2+^, As^3+^/As^5+^, Cr^6+^/Cr^3+^ ions). Monitoring ranges must therefore span from sub-microgram per liter for high-toxicity metals to tens of milligrams per liter for Fe^2+^/Fe^3+^, Mn^2^/Mn^4+^, Ni^2+^, Cu^2+^ ions in industrial streams. Across all sectors, continuous heavy-metal monitoring enhances regulatory compliance, enables rapid response to contamination events, and supports process optimization and environmental protection in ways that intermittent grab sampling cannot [37,66] (Table 2).

**Table 2:** A list of dangerous heavy metals with their sources, their health and environmental effects, and the WHO guideline values for drinking water.

Metal ion	Sources of contamination	Health and environmental effects	WHO guideline value	Reference

lead (Pb^2+^)	corroding pipelines, batteries, industrial effluents, paints, mining	strong neurotoxin; lowers IQ in children; cardiovascular, kidney, and reproductive toxicity	0.01 mg/L	[2,71–72]
mercury (Hg^2+^)	mining, coal combustion, industrial waste, dental/medical waste	severe neurotoxicity; kidney damage; bioaccumulates as methylmercury in aquatic life	0.006 mg/L	[2,71–72]
arsenic (As^3+^)	natural groundwater leaching, mining, pesticides, industrial waste	carcinogenic (skin, bladder, lung); skin lesions; cardiovascular disease; developmental toxicity	0.01 mg/L for total arsenic	[2,71–72]
arsenic (As^5+^)	oxidized groundwater, industrial discharge, agricultural chemicals	less toxic than As^3+^ but still carcinogenic with long-term exposure; associated with skin, bladder, and lung cancers; more strongly adsorbed in soils, making it comparatively less mobile	0.01 mg/L for total arsenic	[2,71–72]
chromium (Cr^6+^)	leather tanning, electroplating, stainless steel industry, pigments	Cr^6+^ is carcinogenic; causes DNA damage, skin irritation, gastrointestinal injury	0.05 mg/L for total chromium	[2,71–72]
chromium (Cr^3+^)	natural minerals, steel industry, leather tanning	essential trace nutrient at low levels, but excessive exposure may cause skin irritation and organ toxicity; considerably less toxic and less mobile than Cr^6+^	0.05 mg/L for total chromium	[2,71–72]
cadmium (Cd^2+^)	mining, batteries, fertilizers, electroplating, waste disposal	kidney damage, bone demineralization, carcinogenic, bioaccumulative	0.003 mg/L	[2,71–72]
manganese (Mn^2+^)	natural geology, mining, wastewater, industrial runoff	essential nutrient but toxic at high levels; neurological impacts; affects childrens’ cognitive development	0.08 mg/L	[2,73]
manganese (Mn^4+^)	oxidized water systems; strong oxidants used in water treatment; industrial discharge	occurs as insoluble MnO_2_ in oxidized waters, less toxic than Mn^2+^, elevated Mn^4+^ particulates can cause discoloration, turbidity, and deposition in pipes, and may release Mn^2+^ under reducing conditions	0.08 mg/L for total manganese	[2,73]
iron (Fe^2+^)	natural soil/rock erosion; corroded iron pipes; groundwater under reducing conditions	not highly toxic but causes aesthetic issues, promotes bacteria, can cause oxidative stress when very high	(Rec) 0.3 mg/L for total iron	[2,66]
iron (Fe^3+^)	oxidized water systems, industrial discharge, corroded infrastructure	less soluble than Fe^2+^ and tends to precipitate as iron oxides; lower direct toxicity, but excessive levels can cause turbidity, pipe scaling, discoloration, and ecological imbalance in aquatic systems	(Rec) 0.3 mg/L for total iron	[2,66]
nickel (Ni^2+^)	electroplating, stainless-steel corrosion, industrial discharge	dermatitis, respiratory problems, possible carcinogenicity; toxic to aquatic organisms	0.07 mg/L	[2,74]
copper (Cu^2+^)	corroded copper pipes, mining, industrial effluents	essential nutrient but harmful at high levels; liver/kidney damage; toxic to fish and aquatic life	2 mg/L	[2,66,72]

### Device geometries

**Figure 1:**
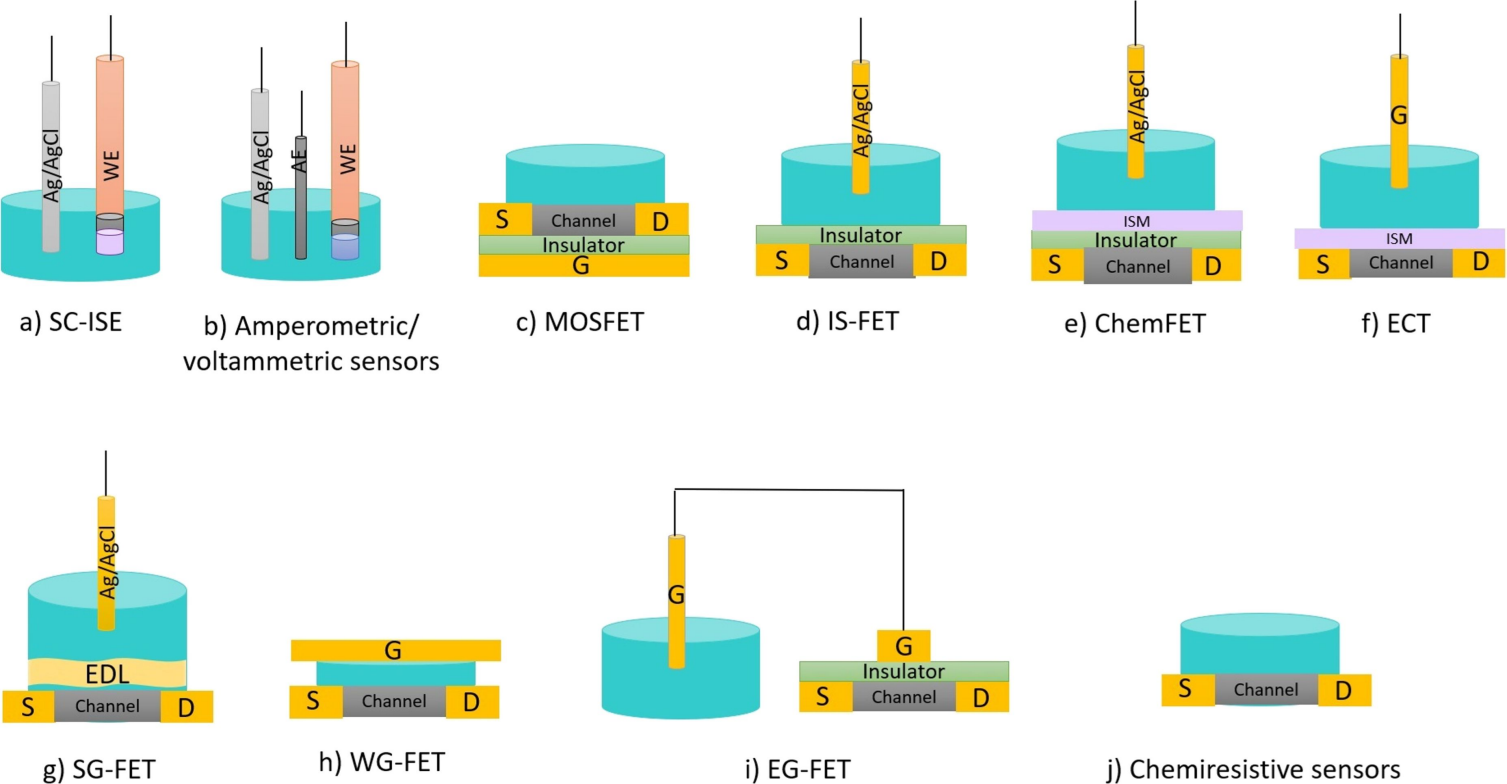
Schematic overview of major electrochemical and electrical sensor geometries for ion detection. (a) Solid-contact ion-selective electrodes (SC-ISEs) with no internal filling solution in working electrode covered with a layer of ISM (ion-selective membrane in purple color; uses solid transducer, (b) amperometric/voltametric sensors, (c) metal-oxide-semiconductor FETs (MOSFETs) with back-gated geometry, (d) ion-sensitive FETs (IS-FETs) which oxide surface directly exposed to electrolyte, (e) chemical FETs (ChemFETs) with ion-selective membrane on top of IS-FET, (f) electrochemical transistors (ECTs) that volumetric ion penetration modulates channel, (g) solution-gated FETs (SG-FETs) with EDL gating at channel–electrolyte interface, (h) water-gated FETs (WG-FETs) in which thin water layer acts as gate dielectric, (i) extended-gate FETs (EG-FETs) with an external sensing electrode connected to the MOSFET gate, and (j) chemiresistive sensors. Used symbols are S = source; D = drain; G = gate; Ag/AgCl = reference electrode; WE = working electrode; AE = auxiliary electrode; ISM = ion-selective membrane; EDL = electric double layer.

To meet the increasing demand for real-time and in situ analysis, electrical and electrochemical sensors have gained prominence owing to their portability, low power consumption, and adaptability to complex aqueous matrices. Several device geometries of potentiometric, voltammetric, amperometric, field-effect transistor (FET)-based, and chemiresistive sensors have been explored for online water monitoring (Figure 1). Each sensor type differs in its transduction mechanism, structural configuration, and material composition, which ultimately influence its analytical performance, stability, and ease of integration into autonomous systems [22].

Potentiometric sensors, particularly ion-selective electrodes (ISEs), remain among the most established technologies for the detection of ionic species in water. As illustrated in Figure 1a, an ISE generally consists of a disk or planar electrode coated with a polymeric ion-selective membrane (ISM) containing an ionophore [75]. In ISEs, the potential changes at the membrane–sample interface against a reference electrode follow the Nernst equation allowing for selective detection of specific ions such as nitrate, potassium, and calcium [76]. For a monovalent ion at 25 °C, the theoretical Nernstian slope is ≈59.16 mV/decade of activity change, decreasing proportionally with temperature and scaling as 59.16/*z* for ions of charge *z* [77]. Modern solid-contact ISEs (SC-ISEs) replace the inner reference solution with a solid electron–ion transducer, typically based on conducting polymers or nanostructured carbon materials [76,78]. SC-ISEs convert the ionic potential formed at the membrane–sample interface into an electronic signal through one of three principal mechanisms: (1) Capacitive (electrostatic) transduction relies on high double-layer or pseudocapacitive charge storage at the solid contact; carbon nanotubes (CNTs) are a widely used material, which provides a large interfacial capacitance and excellent potential stability. (2) Redox (faradaic) transduction uses redox-active materials in which reversible electron-transfer reactions buffer potential changes, for example ferrocene-doped conducting polymers whose Fe^2+^/Fe^3+^ redox couple provides a stable and reproducible interfacial potential. (3) Mixed ionic–electronic conduction occurs in materials such as PEDOT:PSS, which supports simultaneous ion and electron transport and transduces potential changes via modulation of the polymer oxidation state. Together, these mechanisms enable SC-ISEs to achieve stable, Nernstian ion detection without an internal liquid junction. These sensors are cost-effective and energy-efficient for continuous field operation, although potential drift and water-layer formation can affect long-term stability [79]. The requirement for a reference electrode, however creates severe challenges in stability, miniaturization, and long-term maintenance [13]. Solid-contact nitrate-selective electrodes and valinomycin-based K^+^ ISEs integrated on chips for environmental waters are some examples of ISEs [7].

As shown in Figure 1b, voltammetric and amperometric devices share a three-electrode geometry, comprising a working, reference, and counter electrode, often implemented as planar screen-printed electrodes suitable for integration in flow systems. In voltammetry, the applied potential is scanned, and the resulting current response indicates the redox behavior of the analyte [80]. Amperometric sensors, by contrast, operate at a fixed potential and measure a steady-state current proportional to analyte concentration [81]. These sensors are particularly effective for redox-active species or, when coupled with enzymatic films, for indirectly measuring ions such as nitrate or phosphate. While they offer fast response times and high sensitivity, electrode fouling and matrix interference can limit their durability in continuous operation. Anodic stripping voltammetry (ASV) sensors build upon this principle, employing thin-film working electrodes (e.g., bismuth or gold films) that enable preconcentration of trace metal ions before measurement [82]. The ASV process involves two stages: (1) deposition, where metal ions are reduced and plated onto the electrode surface, and (2) stripping, where oxidation peaks are recorded and quantified [82–83]. This approach allows for detection at nanomolar levels and is widely applied for Pb^2+^, Cd^2+^, and Cu^2+^ monitoring in natural waters. The geometry is typically planar and flow-through compatible, but periodic electrode renewal and electrolyte control are necessary for consistent field use [82]. Pb^2+^ and Cd^2+^ determination in natural waters using bismuth-based ASV electrodes is an example of this method for ion detection [84].

Field-effect transistor (FET)-based sensors represent a different class of solid-state devices in which the conductivity of a semiconductor channel is modulated by changes in the surface potential at an electrolyte–gate interface [85]. FET platforms can lead to fast, miniaturized, CMOS-compatible devices (complementary metal-oxide-semiconductor, a common fabrication process for integrated circuit chips) [8–10]. Figure 1c–i illustrates the major FET geometries, each differing in how the gate potential is applied and how the analyte interacts with the channel. It includes metal-oxide-semiconductor FETs (MOSFETs), ion-sensitive FETs (IS-FETs), chemical FETs (ChemFETs), electrochemical transistors (ECTs), solution-gated FETs (SG-FETs), water-gated FETs (WG-FETs), and extended-gate FETs (EG-FETs). In MOSFET-based devices, the gate terminal, typically a metal or another conductive material, is coated with an insulating layer, often the corresponding metal oxide, and the semiconductor channel is exposed to the solution. A gate voltage is externally applied perpendicular to the channel and can be tuned or swept. Interactions between the analyte and the channel modify the electrostatic environment of the channel. This modification alters the channel conductance and changes its dependence on the applied gate voltage. Consequently, variations in the device transfer characteristics can be correlated with the analyte concentration (Figure 1c). An ion-sensitive FET (ISFET) is a modified MOSFET designed so that its insulator (often a metal oxide) on top of the channel is directly exposed to the solution. The electrolyte and a reference electrode form the gate (Figure 1d). The sensor directly responds to surface chemical reactions (protonation/deprotonation of –OH groups) that change oxide surface potential and shift the threshold voltage. Due to this mechanism, highly sensitive and stable IS-FETs for pH monitoring are reported that are CMOS-compatible and can be miniaturized, but this type of FET suffers from poor selectivity to other ions present in water [86–89]. Chemical FETs (ChemFETs or ISM-FETs) incorporate ISMs (containing ionophores) on top of the metal oxide of IS-FETs to solve this problem (Figure 1e). Target ions bind to ionophores in the membrane and generate a membrane potential relative to the fixed potential of a reference electrode, thus modulating the underlying IS-FET threshold voltage. ChemFETs are selective and sensitive devices. For example, Joly et al. developed a ChemFET microsensors to detect NO_3_^−^ and NH_4_^+^ achieving near-Nernstian slopes in liquid and soil phases [90]. It should be considered that incorporating a membrane, as opposed to allowing direct analyte interaction with the sensing surface, tends to increase signal drift due to membrane aging and ionophore leaching, while it can also reduce the sensor’s response speed and its lifetime.

Electrochemical transistors (ECTs) are another type of FETs that translate ionic activity in a solution directly into amplified electronic signals. Their channels are commonly coated with ISMs to increase their selectivity (Figure 1f). ECTs rely on volumetric ion penetration into an active channel (often a mixed ionic–electronic conductor), which modulates its conductivity through redox or doping/de-doping processes. This typically yields high transconductance, strong signal amplification, and excellent sensitivity at low operating voltages, though it can introduce slower response times and long-term drift due to ion trapping or material swelling [91–92]. In contrast, solution-gated FETs (SG-FETs) have a geometry that use an electrolyte as the gate dielectric, and ions modulate the surface potential at the semiconductor–electrolyte interface without penetrating the channel. In these devices, the channel between the source and drain is directly exposed to the analyte solution, and gating occurs through the electric double layer (EDL) that forms at the interface between the channel surface and the electrolyte (Figure 1g). A reference electrode is required to apply the gate voltage, which modulates the EDL capacitance for electrostatic control of the channel. Due to their large EDL capacitance, these devices work well with 2D materials and show ultrahigh sensitivity; they are therefore suitable for determining trace concentrations of heavy metals. The drawback of this device geometry is its susceptibility to contamination and drift as well as its sensitivity to ionic strength fluctuations of the solution (Debye screening) [93–94]. This interfacial gating enables fast, reversible responses and good stability, especially with materials like graphene or CNT networks, but the signal amplification is generally lower because modulation is confined to the surface rather than the bulk. Water-gated FETs (WG-FETs) represent a sub-type of SG-FETs in which a thin layer or droplet of water or electrolyte covers the channel and acts as the gate insulator (Figure 1h). Instead of a reference electrode, a metal electrode is placed in contact with the water layer (or positioned above it) as a gate electrode. This geometry allows for stable operation in aqueous environments and enables continuous monitoring of bulk solution properties, though it similarly faces challenges with interference from electrolyte composition and long-term stability.

Overall, to design a FET sensor for aqueous environments, factors such as ionic strength, fouling, and long-term stability dominate design choices. In electrolytes with realistic ionic strengths, the Debye screening length shrinks to ≈1 nm, which suppresses FET responses to charges located further from the channel; this is the core challenge for liquid-phase FET sensing. Practical routes include lowering ionic strength (when acceptable), using small receptors (aptamers) and porous/charge-excluding coatings (like ChemFETs), nano-confinement, or device formats such as the extended-gate FET (EG-FET) configuration. An EG-FET is a conventional MOSFET device with the gate contact electrically connected to an external sensing electrode, which may consist of glass, Ag/AgCl, or oxide materials, and can also be coated with an ion-selective membrane (Figure 1i). The sensing element is physically separated from the MOSFET, and the potential generated at the external electrode modulates the transistor. A key advantage of the EG-FET architecture is its ability to compensate for environment-induced signal drift. With the EG-FET geometry, the MOSFET is protected from corrosion, and the metal electrode can easily be functionalized or replaced with another electrode. Thus, this device is cost-effective, since it requires only a standard MOSFET and a replaceable electrode, and it offers robustness with the flexibility to easily exchange the sensing head. However, drawbacks include increased parasitic capacitance and higher noise levels due to additional wiring, and the design is not well suited for nanoscale integration [95].

All ion-sensing FET devices inherently require a gate electrode, which is implemented as a reference electrode in many geometries to provide a stable and well-defined electrochemical potential. Incorporating such a reference element brings both advantages and constraints. On the one hand, a stable reference electrode helps decouple the transistor from fluctuations in the sample matrix, improving baseline stability, reducing drift, and enabling more reproducible measurements in complex aqueous environments. This stability is especially valuable when dealing with variable ionic strengths, biofouling, or long-term continuous monitoring, where uncontrolled potential shifts would otherwise dominate the signal [96]. On the other hand, integrating a reference electrode into a compact sensor platform introduces practical challenges. Maintaining a constant potential requires careful material choice, hydration control, and sometimes bulky internal electrolyte reservoirs, all of which complicate miniaturization and packaging. Additionally, adding a separate reference element increases wiring, parasitic capacitance, and mechanical complexity, making it harder to achieve robust, field-deployable devices with consistent performance [29,97].

Chemiresistive sensors, in contrast, use a simpler two-electrode geometry, commonly interdigitated microelectrodes bridged by a conductive sensing film composed of materials such as graphene, reduced graphene oxide, CNTs or conducting polymers (Figure 1j) [11,98–102]. Adsorption or complexation of ions alters the film’s resistance, producing a measurable signal. Selectivity arises from the chemical modification of the sensing layer with specific functional groups or chelating agents [42]. These sensors offer low-cost fabrication, easy scalability, and compatibility with printed and microfluidic systems, although their performance can be affected by baseline drift, and cross-sensitivity to ionic strength [11]. Chemiresistors easily integrate with ISMs, as it was recently reported that membrane-coated devices can achieve low-microgram-per-liter detection of Pb^2+^ in water, hinting at practical continuous monitoring of priority metals [11,103–104].

Each method of detection and sensor geometry has distinct advantages and limitations. Membrane-coated sensors including SC-ISEs, ChemFETs, ECT, and ion-selective chemiresistive sensors are well-suited for long-term, continuous measurements of ion activity due to their selectivity and compact integration. ASV provides high sensitivity for trace metal ions, while amperometric and voltammetric sensors deliver rapid kinetic responses for electroactive analytes [22]. Ongoing advancements in material science and studies on nanostructured interfaces significantly improved the quality of these electronic sensors, which will be discussed in detail in the following sections.

### Active layer materials for electrodes, FETs, and chemiresistors

For effective ion sensing in aqueous media, a channel material for a FET sensor must combine several key attributes. First, high interfacial sensitivity at low voltage, manifested as a large transconductance (µ·C) for thin channels and strong coupling through the EDL in electrolyte gating, is essential for detecting small changes in the ion-induced surface potential. Second, for accurate and reliable ion sensing, the transistor channel material must not chemically degrade or react with the aqueous environment, must minimize charge-trapping-related noise and hysteresis through clean and defect-controlled interfaces, and must maintain stable electronic properties over time so that any electrical signal change reflects only the analyte concentration, not material instability. Third, the material’s surface must allow for functionalization, enabling integration with chelators and/or ion-selective membranes to create a selective binding layer for analyte detection. Finally, processability and scalability of the material are important considerations [105]. For practical ion-sensing platforms, materials must be compatible with mass-producible, reproducible, low-cost, and reliable fabrication techniques. The material choice is therefore influenced not only by performance metrics (sensitivity, limit of detection, selectivity, stability) but also by how easily, inexpensively, and reliably it can be produced, patterned, and integrated into devices at scale. In the case of chemiresistors, the channel should also offer a large accessible surface area to maximize adsorption-induced conductance change. In addition, it should be engineered to operate near the percolation threshold, where charge transport is dominated by a small number of critical junctions, thereby enabling high transduction gain from small adsorption events [106]. The active layer of an electrochemical electrode should provide high electrochemical activity with efficient ion and electron transport, ensuring fast charge/discharge kinetics. It must also maintain chemical stability and mechanical integrity during repeated cycling to deliver long-term performance [107].

#### Inorganic materials

**Semiconducting metal oxides.** Metal-oxide thin films (such as ZnO, SnO_2_, IGZO, In_2_O_3_) are classified as classic inorganic semiconductors with a wide bandgap (3–4 eV). The surface of a semiconducting metal oxide is its most chemically active region that can have a significant impact on the electronic structure of the material. Because metal oxides combine metal cations and oxygen anions with partly ionic bonding, their surfaces behave very differently from covalent semiconductors like silicon. Surface atoms tend to rearrange, react, or adsorb species to reduce surface free energy, leading to non-ideal, defect-rich surfaces. These defects often cause the film to be n-type doped and form surface states inside the bandgap, affecting charge trapping, catalytic activity, and sensing behavior. Due to their structure, charge transfer in metal oxide semiconductor channels is strongly influenced by their defect chemistry, surface adsorbates, and polaronic transport behavior. Charges primarily transfer through defect-induced n-type conduction by a combination of band-like transport and polaron hopping. The charge transfer gets heavily modulated by surface adsorbate interactions and grain boundary barriers. Metal oxides typically show conductivities of 10^−6^–10^2^ S/m, mobilities of 0.1–50 cm^2^/V·s, and electron concentrations of 10^16^–10^19^ cm^−3^. These values shift dramatically with oxygen vacancies, doping, and microstructure. The specific surface area of sensing semiconducting metal oxides (SMOs) is in a range of 20–200 m^2^/g depending on their degree of porosity and nanostructuring [108–109].

**Figure 2:**
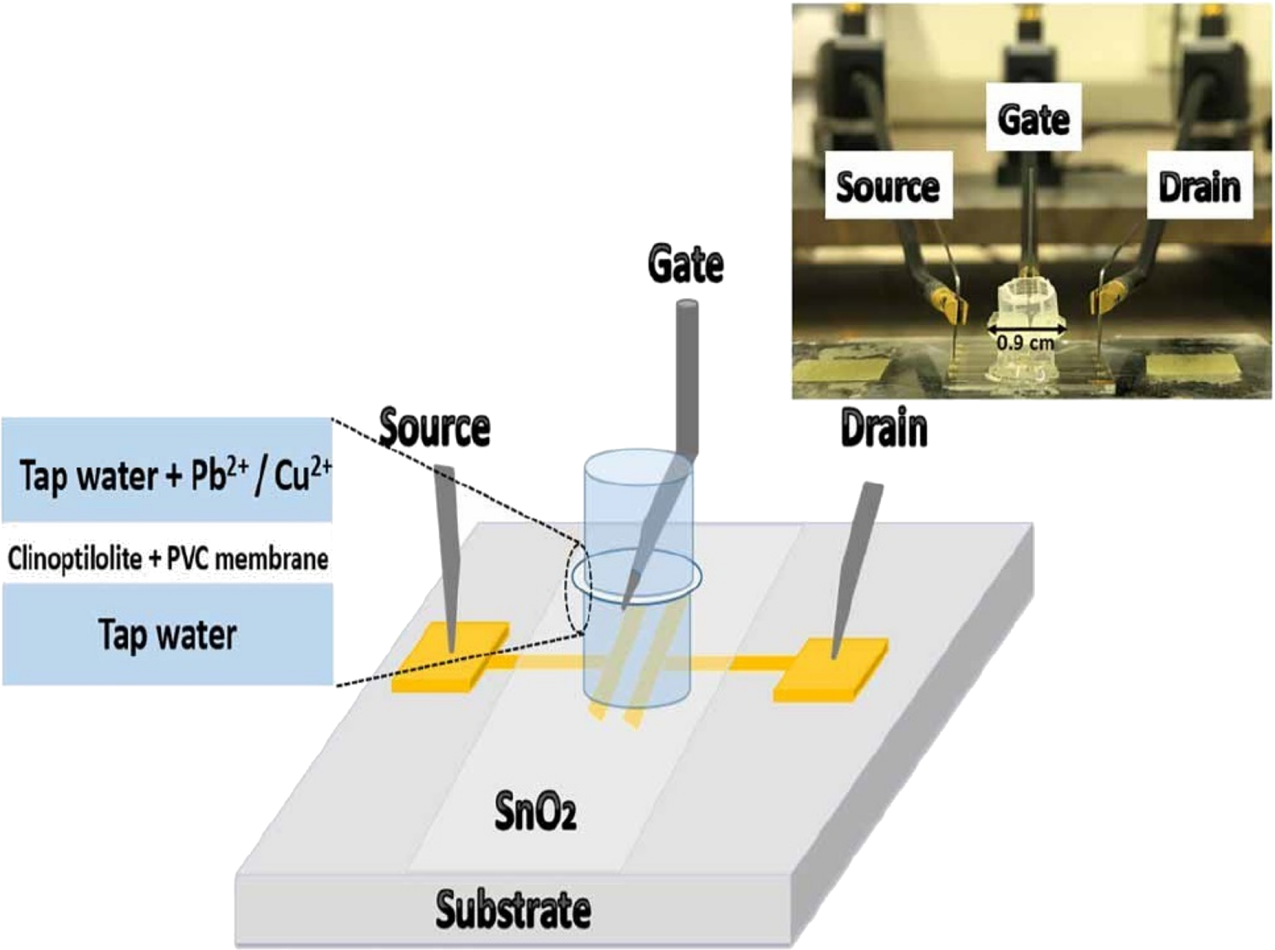
Design of a water-gated field effect transistor sensor. The inner “tap water” pool acts as a reference against the outer “sample” pool. Source and drain transistor contacts are contacted by tungsten needles mounted on Karl Suss probeheads. A third probehead is used to immerse another tungsten needle into the water in the sample pool to provide a gate contact. Inset: photograph of clinoptilolite-sensitised SnO_2_ WG-FET sensor platform. Figure 2 was reproduced from [110] (© 2020 Z. Alqahtani et al., published by IWA Publishing, distributed under the terms of the Creative Commons Attribution 4.0 International License, https://creativecommons.org/licenses/by/4.0).

As an example of an SMO-based device, a WG-FET based on a SnO_2_ thin-film channel was developed for the selective detection of Pb^2+^ and Cu^2+^ ions in water [110]. The device employed a clinoptilolite-filled, plasticized PVC membrane as an ion-selective layer, which generates a potential difference between the sample and reference pools (Figure 2). This potential modulates the threshold voltage (Δ*V*_th_) of the SnO_2_ transistor, with the shift following a Langmuir–Freundlich isotherm that reflects heterogeneous ion-binding behavior. The WG-FET exhibited a rapid response at low ion concentrations and signal saturation at higher levels, achieving detection limits of 0.9 nM for Pb^2+^ and 14 nM for Cu^2+^, both well below the WHO regulatory limits [110]. Because metal-oxide thin films typically exhibit lower conductivity, carrier mobility, and surface area than 2D materials such as graphene or CNTs, integrating them with a highly conductive 2D component allows the hybrid structure to leverage the superior electronic and interfacial properties of the 2D material for effective online ion sensing in electrochemical and electronic devices. Arora et al. demonstrated that combining ZnO with graphene can result in a highly sensitive FET device for Pb^2+^ detection [111]. Integration of conductive graphene with semiconducting ZnO significantly reduces the ZnO bandgap (from ≈3.7 eV to ≈2.2 eV), thereby enhancing charge transfer and overall electrochemical activity. The homogeneous dispersion of nanoscale ZnO on graphene provides a large active surface area for Pb^2+^ adsorption and reduction, resulting in improved sensitivity. Cyclic voltammetry reveals that the redox current varies linearly with the square root of the scan rate, confirming a diffusion-controlled electrochemical process. Additionally, the graphene–ZnO nanocomposite lowers the reduction potential of Pb^2+^ compared to a bare glassy carbon electrode, facilitating electron transfer. Collectively, these effects, bandgap narrowing, increased surface area, and synergistic catalytic activity, make the graphene–ZnO nanocomposite a sensitive and selective film for detecting trace lead ions in aqueous environments [111].

Although metal oxides have good chemical stability and exhibit robust semiconducting behavior with high transconductance, making them good candidates for gas and pH sensing [112–113], their high density of surface states makes sensing in aqueous environments challenging. A high density of surface states can cause charge scattering effects, which decrease the charge mobility while increasing contact resistance, noise, and hysteresis. The FET device can be designed in a way that mitigates these issues, but at the cost of increased complexity. Nonetheless, detection of trace concentrations of heavy-metal ions in real water has already been practically demonstrated by metal-oxide-based electrochemical methods in a much simpler way than the design and fabrication process needed for metal-oxide FET devices. ASV on inexpensive screen-printed electrodes routinely achieves sub-parts-per-billion LODs and has already been packaged for in situ and portable use. For instance, a cost-effective electrochemical sensor based on Fe_3_O_4_/MWCNTs/LSG composites functionalized with chitosan was fabricated for the simultaneous detection of Cd^2+^ and Pb^2+^ using ASV. The in situ bismuth film deposition enabled ultralow detection limits of 0.1 μg/L (Cd^2+^) and 0.07 μg/L (Pb^2+^) [114]. That is why the field of ion sensing by metal-oxide FETs has developed more slowly.

In terms of device fabrication, metal oxides often endure harsh aqueous conditions (acidic and alkaline) better than many organics, making them favorable for long‐term deployment. They have well-established fabrication methods (thin films, sputtering, ALD, solution deposition) [115]. They also face some challenges in sensing; for instance, in high‐ionic‐strength aqueous media, the Debye screening length can be very short (<1 nm). Hence, ion‐to‐channel coupling often relies on an ion‐selective membrane that will be in a ChemFET geometry or a remote gate with a EG-FET geometry rather than direct ion adsorption on the oxide. This complicates device architecture. Fabrication and processing may need high temperatures or vacuum deposition to achieve high‐quality films, which can limit inexpensive, printed deployment [28].

**Graphene.** Single-layer graphene, few-layer graphene (FLG), and bulk graphene differ in structure and properties, which affect their sensing performance. Single layer graphene is extremely surface sensitive because every atom is at the surface. It has a low density of electronic states near its Dirac point; thus, even minor changes in surface charge can shift its Fermi level significantly, making it highly responsive to external stimuli. Molecules or ions with electric dipoles can therefore easily shift the Fermi level electrostatically. However, its predominantly basal-plane surface, limited density of electrochemically active edge sites, and susceptibility to chemical and mechanical degradation often lead to signal instability in liquid-phase sensing. In contrast, FLG, typically comprising two to five stacked graphene layers, provides improved structural robustness and reduced noise while retaining high electrical conductivity. The increased edge density and defect accessibility in FLG facilitate faster electron transfer kinetics and enhanced electrochemical activity, making it especially suitable for electrochemical sensing platforms. Bulk (multilayer) graphene, which approaches graphite-like behavior, offers excellent mechanical stability and ease of fabrication but suffers from reduced surface accessibility, interlayer charge screening, and slower charge-transfer processes, resulting in lower sensitivity. Thus, it can be concluded that FLG represents the optimal compromise between sensitivity, stability, and electrochemical performance for practical sensing applications [116]. FLG typically shows conductivities of 10^4^–10^5^ S/m, mobilities of 1,000–50,000 cm^2^/V·s, and a sheet carrier density of 10^12^–10^13^ cm^-2^. Its specific surface area ranges from 300 to 800 m^2^/g. Graphene conducts via band-like transport of delocalized π-electrons, with extremely high mobility. Charge transfer from adsorbates or ions occurs by electron donation or withdrawal, shifting the Fermi level and modulating carrier density. Hopping transport only occurs if graphene is heavily defected (e.g., reduced graphene oxide, rGO) [117]. Graphene is a strong channel candidate for FET devices because its atomically thin, all-surface conduction enables very good coupling to the electric double layer under electrolyte gating, yielding large transconductance at low bias and high sensitivity to ion-induced interfacial potential changes. In practice, graphene (and rGO) integrates readily with ion-selective membranes, which localize the recognition event within the Debye length and translate ion activity into reproducible shifts in the Dirac point [118].

Chen et al. developed a rGO-based FET sensor for detection of nitrate ions in water. The device employs rGO nanosheets as the conductive channel, functionalized with benzyltriethylammonium chloride (TEBAC), whose quaternary ammonium groups exhibit strong electrostatic affinity for nitrate ions. The TEBAC molecules are immobilized on rGO via π–π interactions, forming a stable hybrid sensing layer. Upon exposure to nitrate, electrostatic binding between NO_3_^−^ and the –N^+^(CH_2_CH_3_)_3_ sites induces electron transfer and redistribution within the rGO/TEBAC interface (since nitrate ions have a delocalized π bond due to the p_z_ orbitals from nitrogen and oxygen atoms, after adsorption onto the TEBAC probe, they interact with the electron cloud of the rGO nanosheets), leading to a modulation of carrier density and a measurable decrease in channel conductance (due to the fact that rGO material is p-doped). This transduction process enables the device to rapidly (2–7 s) achieve an ultralow limit of detection (4.9 µg/L NO_3_^−^) with excellent selectivity against competing anions (Cl^−^, SO_4_^2−^, CO_3_^2−^). The sensor’s performance, reusability through simple NaCl regeneration, and enzyme-free design underscore its potential as a robust, low-cost platform for real-time nitrate monitoring in water [119].

**Figure 3:**
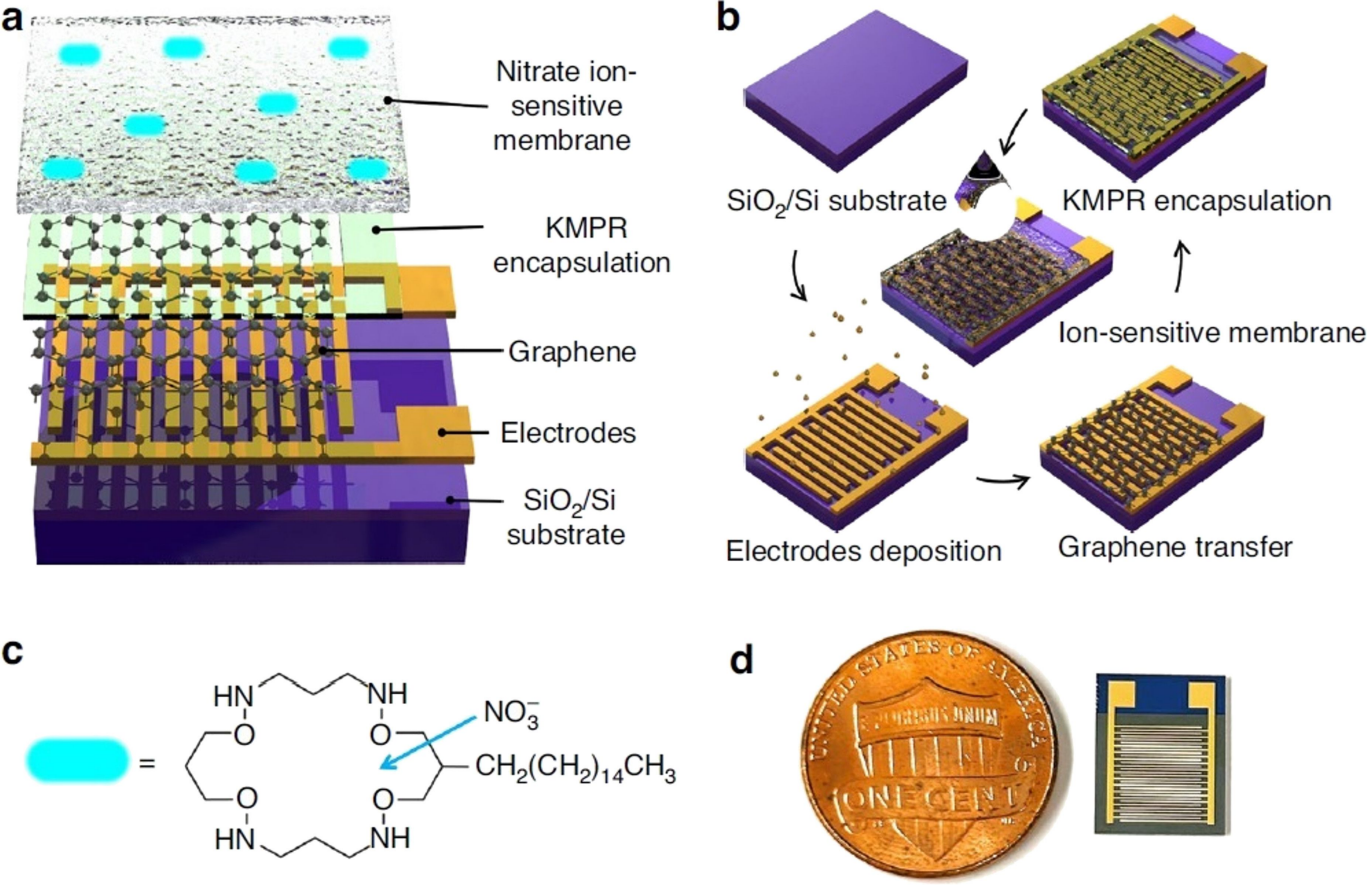
Structure and fabrication of graphene ion-sensitive field-effect transistors (ISFETs). (a) Schematic illustration of the structure of the devices. (b) Fabrication flow chart. Preparation of SiO_2_ substrate. E-beam deposition of Cr/Au source-drain electrodes. CVD graphene transfer. Device encapsulation via KMPR negative photoresist. Device dicing and a nitrate ion sensitive membrane deposition. (c) Chemical structure of nitrate ionophore. (d) Optical image of an individual graphene ISFET beside of one cent coin. Figure 3 was reproduced from [17] (© 2024 Y. Xu et al., published by Springer Nature, distributed under the terms of the Creative Commons Attribution 4.0 International License, https://creativecommons.org/licenses/by/4.0).

Also, ultratrace nitrate detection was proposed with an interdigital graphene-FET integrated with a nitrate-sensitive membrane (Figure 3). The sensing mechanism relies on selective complexation of nitrate ions with ionophores within the ISM, which generates a membrane potential proportional to the nitrate concentration according to the Nernst relation. This potential modulates the graphene surface potential, shifting its Fermi level and producing a measurable Dirac point displacement (Δ*V*_Dirac_) in the transfer characteristics. Optimization of the graphene channel geometry and ISM thickness enhances the device transconductance, amplifying its response to ionic potential changes. The resulting electrical signal, expressed as a linear Dirac point shift with logarithmic dependence on nitrate concentration, enables precise quantitative detection with a low limit of detection (0.041 ppt; 4.8 × 10^−13^ M), a wide linear range (0.1 ppt to 100 ppm), and Nernstian sensitivity (≈28 mV/decade), with excellent selectivity against common anions and validated performance in real water samples [17].

Graphene’s processability as inks and large-area films also supports scalable arrays and simple chemiresistive formats, further underscoring its suitability for practical sensing application [11]. Despite graphene’s advantages, it exhibits several intrinsic limitations that can hinder its effectiveness as a FET channel material. Its zero bandgap results in intrinsically low on/off current ratios, high off-state leakage, and excessive static power waste, preventing reliable digital switching performance. Efforts to induce a bandgap, such as graphene nanoribbons, bilayer gating, or strain engineering, inevitably degrade mobility and increase scattering. Device performance is further limited by strong sensitivity to substrate interactions (e.g., charge traps, phonon scattering, and impurities on SiO_2_), which introduce noise and variability. High and inconsistent metal–graphene contact resistance, arising from Fermi-level pinning and the absence of a semiconducting gap, also hampers reproducible device operation. Moreover, fabrication processes often leave polymer residues and adsorbates that deteriorate electronic quality, and large-area CVD graphene suffers from structural non-uniformities, including grain boundaries, wrinkles, and non-uniform doping, that create substantial device-to-device variability [120]. Beyond these limitations, studies found how graphene’s high mobility, chemical functionalizability and compatibility with extended-gate layouts help overcome screening and drift in complex electrolytes and make it suitable for electrical and electrochemical sensing [117,120–121].

**Carbon nanotubes.** Carbon nanotubes (CNTs) are one-dimensional nanostructures composed of seamless cylinders of sp^2^-bonded carbon atoms formed by rolling up graphene sheets, with their material properties governed primarily by the number of walls, diameter, chirality, and defect structure. Single-walled carbon nanotubes (SWCNTs) consist of a single graphene cylinder with complete surface exposure and a highly anisotropic, chirality-dependent electronic structure with either metallic (zero bandgap) or semiconducting (a bandgap of 0.5–1.5 eV) character, resulting in favorable intrinsic electrical and mechanical properties but relatively low robustness. Double-walled carbon nanotubes (DWCNTs), comprising two concentric graphene shells held together by van der Waals interactions, exhibit a hierarchical architecture in which the outer wall dictates surface chemistry while the inner wall enhances mechanical integrity and stabilizes charge transport. Multiwalled carbon nanotubes (MWCNTs) are composed of multiple concentric shells or a scroll-like structure, display predominantly metallic behavior, and outstanding mechanical strength and chemical durability, though with reduced surface accessibility of inner walls. Across all CNT types, SWCNTs are ideal for ultrasensitive electrical detection, DWCNTs provide a balanced combination of performance and stability, and MWCNTs are particularly well-suited for robust, scalable electrochemical sensors and composite-based electrodes [122]. CNTs typically show conductivities of 10^4^–10^6^ S/m, mobilities of 10,000–100,000 cm^2^/V·s, and charge carrier densities of 10^15^–10^19^ cm^−3^. CNTs possess high specific surface area (SSA; generally in the range of 200–800 m^2^/g) and can be functionalized with selective ionophores, membranes, or chelating ligands [20]. In CNTs, charges move inside individual CNTs via band conduction, then cross to another tube via tunneling/hopping, forming a percolation network. Modulation of the carrier density of CNTs can happen via direct charge transfer, dipole fields (electrostatic gating effect), or Schottky-barrier changes caused by interacting molecules or ions. CNTs are well suited as channel materials for sensing in aqueous environments because their high aspect ratio, one-dimensional structure, and excellent electrical properties yield strong coupling to surface and interfacial phenomena under electrolyte gating [123–124].

Melzer et al. reported the development of a membrane-coated CNT-based electrochemical charge-transfer device for detecting K^+^, Ca^2+^, and Cl^−^ in complex sample matrices with high ionic strength. Upon interaction of a membrane-coated CNT film (semiconducting SWCNT) with the ion of interest, the ion-activity-dependent electrochemical response of polymeric ion-selective membranes modulate the effective gate potential and produce threshold voltage (*V*_th_) shifts in the FET transfer characteristics. Potassium sensing using a valinomycin-based ISM yielded a linear *V*_th_ shift of +39.3 mV per decade of K^+^ activity from 10^−1^ to 10^−7^ M. A chloride-selective membrane incorporating tridodecylmethylammonium chloride produced a −51.5 mV/decade shift between 10^−1^ and 10^−5^ M, while calcium sensing using a diamide-based membrane achieved a +21.7 mV/decade response from 10^−1^ to 10^−6^ M with good selectivity over common interfering ions [125].

Although SWCNTs exhibit high intrinsic carrier mobility and electrostatic gate control, they face several challenges. A major challenge is the presence of mixed metallic and semiconducting CNTs in as-grown films, which may degrade device performance by lowering on/off ratios and causing leakage pathways that are difficult to eliminate at scale. Even for semiconducting SWCNTs, electronic properties are highly sensitive to chirality, diameter, and defects, leading to substantial device-to-device variability [126]. CNT networks also suffer from high contact resistance at metal–nanotube and tube–tube junctions, which limits current drive and introduces nonuniform conduction pathways [127–128]. Moreover, environmental sensitivity, particularly the adsorption of oxygen and other ambient contaminants, can lead to threshold-voltage instability and hysteresis, thereby complicating biasing conditions and degrading long-term reliability [129]. Despite these challenges, many CNT sensors for water-based analytes have been reported, emphasizing that CNT films can deliver good stability, low baseline drift and robust responses when properly engineered for aqueous operation [20,130–131].

**Silicon nanowires.** Silicon nanowires (SiNWs) consist of silicon confined to nanoscale diameters (10–100 nm) and elongated to form high-aspect-ratio one-dimensional structures. The bandgap of bulk silicon is 1.12 eV. Very thin SiNWs (<5–10 nm) have a larger bandgap due to quantum confinement (up to ≈2 eV) depending on their diameter. SiNWs behave similarly to doped bulk silicon, but surface depletion and quantum confinement decrease their conductivity (10^2^–10^4^ S/m) and mobility (50–1500 cm^2^/V·s). Charge transport in SiNWs is primarily band-like inside the crystalline core. Even though surface effects reduce conductivity and mobility, they dramatically enhance sensitivity since surface depletion means surface charge changes control the entire wire. Quantum confinement results in strong current modulation by even small potential changes. This is why SiNWs are among the most sensitive FET-based chemical sensors. Their charge carrier density is 10^15^–10^18^ cm^−3^ and their SSA falls in the range of 10–100 m^2^/g, depending on diameter and aspect ratio. Work on silicon SiNW-FETs has shown that binding of charged ions at the solid–liquid interface leads to measurable shifts in threshold voltage or channel conductance, enabling label-free detection and rapid responses [132–134]. Charge transfer between SiNWs and analyte species occurs through electrostatic gating by intrinsic charges, direct electron donation/withdrawal by the analyte, and trap-mediated exchange at oxide surface states. The native and engineered oxide surfaces on silicon allow for precise functionalization (e.g., via silane chemistry and self-assembled monolayers) to install ion-selective membranes [135–136]. A study by Chen et al. demonstrates an integrated sensing platform based on SiNW-ChemFETs capable of detecting sodium ions. Selectivity toward Na^+^ is achieved through a membrane deposited on the gate insulator, where the Na-ionophore III selectively complexes Na^+^, generating a stable interfacial potential at the membrane–electrolyte boundary that modulates the SiNW channel and produces a potentiometric response. This ion-coupled gating mechanism enables the sensor to exhibit a near-Nernstian sensitivity of 57.9 ± 0.7 mV/decade across concentrations from 100 µM to 100 mM, with a detection limit of ≈60 µM, matching the performance of conventional ion-selective electrodes employing the same ionophore. Despite its strong intrinsic selectivity, the membrane suffers from competitive binding effects, particularly from K^+^, yielding a selectivity coefficient of 10^−1.2^ for Na^+^ over K^+^. This interference effects become pronounced in complex matrices such as river water, though their influence diminishes at higher Na^+^ activities. Thus, further membrane optimization is needed to mitigate molecular-ion partitioning and enhance performance in real-world environmental samples [137].

SiNWs offer good electrical stability and noise characteristics when properly passivated and have attracted significant interest as channel materials for FETs; however, several key limitations hinder their broad practical adoption. The high surface-to-volume ratio of SiNWs causes their electrical performance to be heavily dominated by surface states and interface traps, resulting in degraded carrier mobility and pronounced hysteresis in transfer characteristics [134,138–139]. Additionally, quantum confinement and stochastic dopant distributions in extremely thin SiNWs introduce variability in threshold voltages, making reproducibility difficult across large arrays [140]. Similar to other materials, when SiNW-based sensors are used in high‐ionic‐strength environments, Debye screening and drift of the reference electrode are possible, which further limit robustness and real‐world applicability [141]. In terms of device fabrication, SiNW-FETs are a commercially proven, industrially scalable sensing technology, ideal for miniaturized, mass-produced, electronics-integrated ion sensors; however, achieving uniform nanowire diameter, high crystalline quality, low-resistance contacts, and conformal coating of the channel with a highly dielectric insulating material in the fabrication process still are of concern to researchers in this field [136,141].

**Aluminium gallium nitride/gallium nitride heterostructures.** An aluminium gallium nitride/gallium nitride (AlGaN/GaN) heterostructure is a layered semiconductor made by placing AlGaN (with a bandgap of 3.4–6.2 eV) on GaN (with a bandgap of 3.4 eV), creating a 2D electron gas (2DEG) at the interface due to the strong spontaneous and piezoelectric polarization fields inherent to the wurtzite crystal structure of group-III nitrides. When a thin AlGaN barrier is grown on GaN, the discontinuity in polarization charges creates an intense internal electric field that bends the GaN conduction band downward, forming a triangular quantum well at the heterojunction. As a result, charge transport in AlGaN/GaN devices is dominated by this 2DEG layer, which serves as the conductive channel in high-electron-mobility transistors (HEMTs) with minimal impurity scattering. 2DEG in AlGaN/GaN structures typically shows conductivities of 10^4^–10^5^ S/m, mobilities of 1000–2000 cm^2^/V·s, and electron densities of 10^12^–10^13^ cm^−2^ in the 2DEG. The 2DEG’s proximity to the surface (20–30 nm below the AlGaN barrier) also makes it very sensitive to surface charges, enabling strong field-effect modulation by adsorbates, ions, or electrochemical interactions. Charge transfer between analyte species and the channel occurs electrostatically; external charges or dipoles modulate the 2DEG density by changing the surface potential of AlGaN. Direct charge transfer is weak because GaN is chemically inert, but surface traps can capture or release charge, altering band bending and 2DEG conductivity [142]. Zhao et al. introduced a differential extended-gate (DEG) AlGaN/GaN HEMT device (a type of EG-FET geometry) for detection of Fe^3+^ ions in aqueous environments. GaN (measuring gate) is capped with a layer of gold, which serves as the surface for a self-assembled monolayer of 2-mercaptosuccinic acid. The thiol groups in 2-mercaptosuccinic acid form Au–S linkages with the gate surface, while the carboxyl groups selectively bind Fe^3+^ ions. Therefore, the surface charge modulates at the measuring gate and generates localized charge variations. These changes are capacitively coupled to the HEMT channel, altering the density of the 2DEG and producing a measurable shift in the output signal. The differential configuration, comprising a reference and a sensing gate, cancels common-mode noise from environmental fluctuations such as light or vibration, thereby enhancing detection precision. This device achieved a very low detection limit of 10 fM, a broad dynamic range (10 fM–100 mM), and excellent linearity (*R*^2^ = 0.9955), outperforming conventional AlGaN/GaN HEMT sensors and demonstrating strong potential for real-time monitoring of trace concentration of ions [95].

The 2DEG at the interface delivers high transconductance at low bias while remaining chemically and physically stable in aqueous electrolytes, resulting in low-noise AlGaN/GaN HEMT devices [143–146]. Selective detection of PO_4_^3−^ using ion-imprinted polymers (IIPs), as well as quantification of NO_3_^−^, Hg^2+^, Ca^2+^, and Pb^2+^ ions via ISMs have also been demonstrated with AlGaN/GaN HEMT devices, illustrating the platform’s compatibility with ion-selective layers for specificity [147–151]. The architecture also supports water-gated and extended-gate formats [95,152]. In terms of scalability of these transistors, GaN-on-Si or GaN-on-sapphire epitaxy allows for full wafer-scale growth using metalorganic chemical vapor deposition (MOCVD), compatible with power-electronics fabrication workflows. Batch processing enables thousands of devices per wafer. GaN HEMTs are already manufactured at scale for radio frequency and power electronics, meaning the industry infrastructure already exists. The wide bandgap and chemical stability also make them suitable for aqueous ion sensing directly in harsh environments without special lab-only handling. Meanwhile, performance and reliability of AlGaN/GaN transistors in sensing applications are hampered by several device-level challenges, including self-heating effects, current collapse, gate leakage, high electric field concentration, elevated ohmic contact resistance, and threshold voltage instability. Excessive channel heating and charge trapping can degrade sensitivity and stability, while gate leakage and high contact resistance reduce power efficiency and transduction accuracy. Moreover, threshold voltage drift and field-induced trapping compromise long-term reliability and reproducibility of sensor responses. To overcome these limitations, optimization of thermal management, suppression of trapping effects, and minimization of leakage and contact resistance are essential. Incorporating advanced structural strategies, such as field plates, passivation layers, and refined gate architectures, can further improve the stability, sensitivity, and overall performance of AlGaN/GaN-based sensors [142].

**MXenes.** MXenes (e.g., Ti_3_C_2_T*_x_*) are metallic 2D transition-metal carbides/nitrides with abundant surface terminations (–O, –OH, –F, generated during the etching process using hydrochloric acid and lithium fluoride), which are represented by T*_x_* with a fractional coverage of *x*. Their metal centers possess partially filled d orbitals with a high density of states at the Fermi level and strong M–C or M–N bonding, which leads to delocalized electronic states, high electrical conductivity (10^4^–10^5^ S/m), and small or zero bandgaps (0–0.3 eV). Electrons move freely within the 2D basal plane, similar to a disordered metal. MXenes are formed by wet-chemical etching. They carry functional groups on their surfaces, which cause electron scattering, local band bending, and modification of the effective mass of the charge carriers. This limits mobility (1–100 cm^2^/V·s) and prevents fully ballistic transport. In the films, interflake transport occurs via tunneling and hopping, which influences the conductivity of the film. Thus, thin flakes conduct better than thick, restacked films. In MXenes, ions (H^+^, Li^+^, Na^+^, K^+^, Ca^2+^) can enter the interlayer space and change the charge distribution, resulting in mixed ionic–electronic transport. Few-layer MXenes (delaminated form) typically show a SSA of about 50–300 m^2^/g, which can be modified by engineering the structure such as making it porous or pillared. Due to the metallic d-band contribution from the transition metal centers, MXenes exhibit very high charge carrier densities (10^13^–10^15^ cm^−2^) [153–154].

MXenes exchange charge with their environment via direct chemical electron transfer, electrostatic gating by ions and dipoles, redox reactions involving surface transition-metal sites, and ion-intercalation-induced modulation of the Fermi level. Their exposed metallic surfaces and tunable terminations make them very sensitive to charge-transfer processes. In practice, MXene channels have delivered selective heavy-metal detection in challenging matrices [155–156], for example, Hg^2+^ sensing in high-salinity water using Ti_3_C_2_T*_x_*-based MOSFET devices. The sensor employs Ti_3_C_2_T*_x_* nanosheets as the conductive channel material deposited on interdigitated gold electrodes. The sensing mechanism is governed by the strong adsorption and subsequent reduction of Hg^2+^ to Hg^+^ on the MXene surface, which perturbs the electronic structure. Ti_3_C_2_T*_x_* nanosheets are electron-rich, so when they donate electrons to mercury ions, the channel conductivity decreases, resulting a drop in current. This intrinsic adsorption–reduction process enables high sensitivity and selectivity toward Hg^2+^, with negligible interference from competing metal ions (As^5+^, Cd^2+^, K^+^, Pb^2+^, Cu^2+^, Ca^2+^). Furthermore, the device maintains stable performance in 1 M NaCl, demonstrating robustness against interfering species [157].

MXenes also interface well with ion-selective layers [12], and MXene-based FETs can be fabricated via printing or solution-based methods [158]. A limitation to the application of these materials is their ease of oxidation in aqueous solutions, but recently proposed chemistry and storage strategies (defect passivation, polymer composites, controlled dispersions) address this issue and extend operational lifetimes [153–154]. Therefore, the high conductivity, hydrophilicity, tunable surface terminations, and processability of MXenes make them a suitable choice for sensing applications.

**Transition metal dichalcogenides.** Transition metal dichalcogenides (TMDCs) are a broad class of layered materials with the chemical formula MX_2_, where M is a transition metal (e.g., Mo, W, Nb, V, Ti) and X is a chalcogen (S, Se, Te). In their bulk form, TMDCs consist of stacks of weakly bonded van der Waals layers, each only a few angstroms thick, allowing individual monolayers to be mechanically exfoliated or chemically isolated. Each monolayer comprises a plane of metal atoms sandwiched between two planes of chalcogen atoms, forming a trigonal prismatic or octahedral coordination environment. TMDCs show conductivities of 10^−3^–10^2^ S/m, mobilities of 0.1–500 cm^2^/V·s, and SSAs in the range of 10–200 m^2^/g (few-layer, exfoliated nanosheets) [159–160]. The electronic band structure of TMDCs is strongly thickness-dependent. In the bulk form, interlayer coupling leads to an indirect bandgap (1.0–1.3 eV), whereas removal of interlayer interactions and enhanced quantum confinement in the monolayer shift the band extrema to the same k-point, yielding a larger direct bandgap (1.6–2.1 eV). This well-defined, thickness-tunable bandgap allows for strong electrostatic gate control over carrier transport, which is essential for efficient transistor switching [161–162]. TMDCs conduct charge via band conduction in high-quality monolayers, trap-limited transport in most practical devices (made with CVD-grown TMDCs), and hopping/tunneling in defective or multilayer films (solution-processed TMDC nanosheets, printed electronics, and thick films). Transport is strongly influenced by defects, the dielectric environment, and contact interfaces [163–164].

TMDCs undergo strong chemical charge transfer with adsorbates, electrostatic coupling with external charges, and trap-mediated exchange at vacancies. Their atomic thickness makes their conductivity extremely sensitive to molecular doping, contact barriers, and ionic environments, enabling powerful sensing capabilities [165]. An example of a sensing application of TMDCs is an agent-free MoS_2_-based FET sensor that was developed for real-time and in situ monitoring of silver ions in aqueous media. Agent-free means that MoS_2_ acted as active layer and sensing layer without any additional recognition agents. In MoS_2_, surface sulfur atoms interact with Ag^+^ ions; Ag^+^ acts as a Lewis acid, accepting lone-pair electrons from S atoms and inducing p-type doping. This adsorption decreases electron concentration in the MoS_2_ channel, resulting in a positive threshold voltage shift, reduced carrier mobility, and lower the channel current. The sensor achieved an ultralow detection limit of 10^−10^ mol·L^−1^ and rapid response (≤5 min) with high operational stability [166].

**Figure 4:**
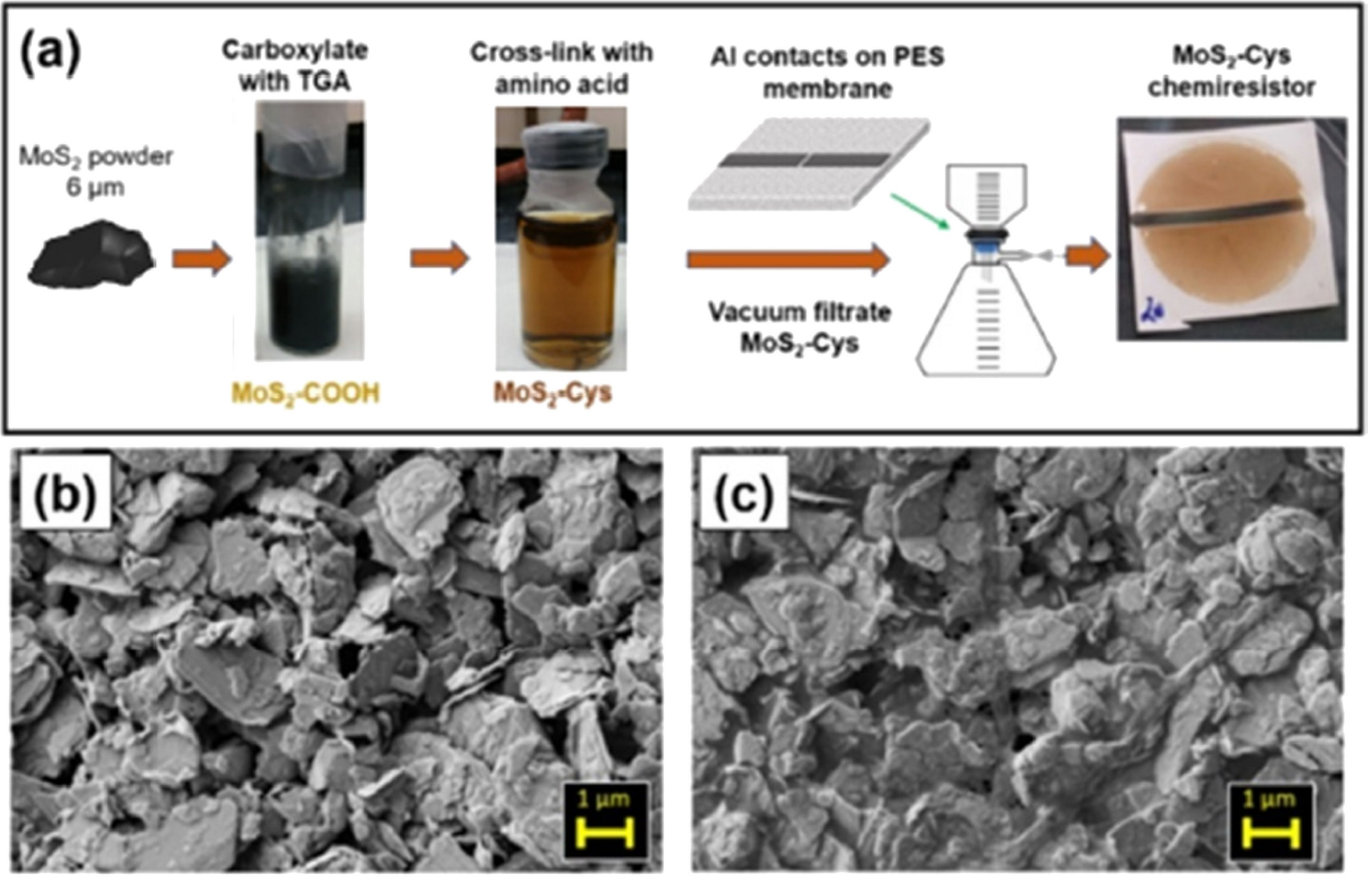
(a) MoS_2_−Cys preparation using thioglycolic acid (TGA) to add carboxyl groups followed by amide cross-linking for Cys functionalization and fabrication of two-terminal chemiresistors. MoS_2_−Cys thin films can be prepared by vacuum filtration and incorporated in these devices. 2 keV scanning electron microscopy (SEM) images of (b) MoS_2_−COOH and (c) MoS_2_−Cys (1:20 MoS_2_/Cys mass ratio), showing Cys functionalization as a network of filaments on the surface of MoS_2_ aggregates. Figure 4 was reprinted from [167], P. Bazylewski et al., “Solid-State Chemiresistors from Two-Dimensional MoS_2_ Nanosheets Functionalized with ʟ-Cysteine for In-Line Sensing of Part-Per-Billion Cd^2+^ Ions in Drinking Water”, *ACS Omega*, Copyright © 2019 American Chemical Society, licensed under the Standard ACS Author Choice/Editors’ Choice Usage Agreement, https://pubs.acs.org/page/policy/authorchoice_termsofuse.html. This content is not subject to CC BY 4.0.

Another study reports the development of solid-state chemiresistors based on MoS_2_ nanosheets functionalized with ʟ-cysteine (Cys) for real-time detection of cadmium ions (Cd^2+^) in drinking water at ultralow concentrations (1–500 ppb) (Figure 4). The MoS_2_-Cys films were fabricated via a simple one-pot synthesis involving thioglycolic acid carboxylation and amide coupling with ʟ-cysteine, deposited onto porous poly(ether)sulfone membranes to enable continuous flow sensing. The sensing mechanism is based on selective adsorption of Cd^2+^ ions to the ʟ-cysteine functional groups, followed by charge transfer that alters the electronic gap states and modulates MoS_2_ conductivity. This interaction results in a substantial resistivity increase, up to 20× at 5 ppb Cd^2+^, with high selectivity under neutral pH conditions. The devices exhibit rapid response (≈1 s), reusability, and operational simplicity without the need for external reagents or complex instrumentation [167].

From a practical perspective, 2D-TMDCs can be fabricated via scalable methods (CVD, ALD, mechanical exfoliation) and integrated into thin-film FET stacks, enabling compact, low-voltage sensors suitable for remote or continuous monitoring of water. They exhibit high mechanical flexibility, chemical stability, and atomically clean surfaces without dangling bonds, making them compatible with a wide variety of substrates, including flexible and transparent electronics. While some challenges remain (such as managing aqueous degradation or selecting optimal functionalization chemistry), the suite of morphological, electrical, and chemical features makes 2D-TMDCs a suitable material class for ion sensing [160].

#### Organic materials

**Small organic molecules and conjugated polymers.** Organic channel materials for organic FETs (OFETs) include conjugated small molecules (such as pentacene, rubrene), and conjugated polymers (such as poly(3-hexylthiophene) (P3HT)- and diketopyrrolopyrrole (DPP)-based polymers). Charge transport in organic semiconductors is fundamentally different from that inorganic materials. Charge transport in organic systems arises from localized electronic states, where carriers move through hopping or polaron mechanisms. Because these materials are held together by weak van der Waals forces, their π orbitals do not form extended bands, and transport is strongly affected by intermolecular arrangement, variations in local site energies, and electrolyte interactions in solution-gated OFET geometry. Charge transport occurs via thermally activated hopping between localized states, resulting in strong temperature dependence, low mobility, and high sensitivity to morphology and crystallinity. Charges in organic semiconductors also distort the surrounding molecular environment, forming polarons that hop between sites at rates governed by reorganization energy and π–π stacking, further limiting mobility and making transport highly sensitive to structural order [168–170].

Organic thin films are structurally heterogeneous, consisting of crystalline π–π stacked domains, amorphous regions, and grain boundaries. Charge transport therefore proceeds along percolation pathways through ordered domains and incomplete percolation leads to drastic drops in charge carrier mobility [168–170]. As highlighted by Lamport et al., the choice of the organic semiconductor strongly impacts charge carrier mobility, on/off ratio, and threshold voltage behavior [168]. In practice, small organic molecules and conjugated polymers used in ECT channels typically exhibit low conductivities (10^−8^–10^−3^ S/m), moderate mobilities (10^−3^–10 cm^2^/V·s), and charge carrier densities of 10^13^–10^18^ cm^−3^, reflecting their localized π electron structure and hopping-dominated transport. Their SSAs are relatively small (1–20 m^2^/g), and their surface-to-volume ratios range from 10^5^ to 10^6^ m^−1^, depending on film thickness and morphology. Upon environmental exposure, charge transfer can occur via electrostatic double-layer gating, electrochemical (Faradaic) charge transfer at higher gate voltages, or trap-mediated exchange [168–170]. For example, Schmoltner et al. presented a modular solution-gated OFET platform for selective, low-voltage ion sensing in aqueous environments. By directly interfacing a P3HT semiconductor with an electrolyte rather than a solid dielectric, the device enables potentiometric detection through a sodium-selective ISM that establishes a Nernstian membrane potential dependent on analyte ion activity. The sensor exhibits a linear, reversible response to Na^+^ concentrations from 10^−6^ to 10^−1^ M with a sensitivity of ≈62 mV/decade and minimal cross-interference from K^+^. The architecture supports straightforward ion exchange for multianalyte detection and demonstrates good operational stability with low drift [171].

Most small-molecule or polymeric organic semiconductors are chosen as the active layer in OFET-based sensors because their molecular structure can be tailored to interact with analytes, and because their fabrication is compatible with flexible substrates and low-cost manufacturing [170,172]. The key merits are ease of chemical modification (to attach recognition groups), compatibility with printable electronics, and sufficient electronic performance for transistors.

**Organic mixed ionic–electronic conductors.** Organic mixed ionic–electronic conductors (OMIECs) are conjugated polymers that enable both ionic and electronic transport, making them ideal for aqueous environments and organic electrochemical transistors (OECTs). Unlike solution-gated OFETs, which rely on surface channels, OMIECs operate through volumetric conduction where ions and electrons move together. Electronic transport occurs via hopping, π–π percolation, and polaron drift, with mobilities of 10^−3^–1 cm^2^/V·s and conductivities rising from 10^−6^–10^−2^ S/m (undoped) to 1–100 S/m (doped) upon electrochemical oxidation. Ionic transport, slower but essential, involves ion diffusion through solvated sites and polymer free volume, with mobilities of 10^−8^–10^−6^ cm^2^/V·s. Ions act as dopants, dynamically modulating conductivity by activating or deactivating electronic states. Because ions permeate the entire film thickness, OMIECs exhibit high volumetric capacitance and strong signal amplification at low voltages. Charge transfer is governed by electrochemical doping, where ionic motion maintains electroneutrality and couples directly with electronic carriers, defining the key performance of OECTs [173]. OMIECs include poly(3,4-ethylenedioxythiophene) (PEDOT) derivatives, glycolated conjugated polymers, DPP-based polymers, redox-active polymers such as polyaniline (PANI), polypyrrole (PPy), and side-chain-engineered polythiophenes. These materials are intrinsically well-suited to aqueous sensing because of their hydrated matrices, which enable volumetric ion uptake and doping/de-doping, yielding large transconductance at low voltage in solution-gated formats. Their ability to swell in water allows for bulk channel participation [173–174].

Among them, poly(3,4-ethylenedioxythiophene) polystyrene sulfonate (PEDOT:PSS) is the most widely used in OECTs as the combination of PEDOT with the polyanion PSS provides solubility, mixed ionic–electronic conduction, and environmental stability. While PEDOT is intrinsically conductive, it is insoluble and requires the sulfonate groups of PSS to maintain its oxidized, p-doped state and to enable dispersion into water, yielding uniform, printable thin films. The resulting composite supports simultaneous electronic transport through PEDOT backbones and ionic transport through the hydrophilic PSS matrix, giving rise to high volumetric capacitance and strong ionic–electronic coupling essential for high-transconductance sensing. PSS also promotes a favorable film morphology by spacing PEDOT chains into a nanostructured network that improves flexibility, mechanical robustness, and tunable conductivity. Furthermore, the PEDOT:PSS ratio can be adjusted to modulate ion penetration, swelling, softness, and device performance. The hydrophilicity and biocompatibility imparted by PSS make PEDOT:PSS stable in aqueous and physiological environments, in contrast to the hydrophobic and unstable pristine PEDOT, thereby positioning it as an ideal material for electronic and aqueous sensing applications [173,175]. For instance, ion-selective OECTs that couple PEDOT:PSS channels to ISMs deliver selective K^+^ and Na^+^ sensing in real time and can be multiplexed or even made on paper substrates for scalable, in-line monitoring [176–178]. Materials innovations (such as porous and foam PEDOT:PSS, as well as tailored side-chain chemistries) further boost transconductance and speed for aqueous detection [179]. Beyond transistors, PANI and PPy offer robust, water-stable chemiresistive channels beneath ISMs; their redox-active backbones and affinity for metal-ion complexation have been leveraged for heavy-metal sensing in water [180]. Finally, OECTs can serve as on-site amplifiers for electrochemical ion sensors, improving sensitivity without complex external electronics, useful for embedded, online analysis in water networks [181].

Analyte detection in OECTs typically occurs through three mechanisms: (1) electrochemical reactions, where analytes oxidize or reduce the polymer; (2) ion uptake, which alters local ion concentration and doping levels; and (3) pH or ion-specific modulation, where species such as H^+^, Na^+^, K^+^, or Ca^2+^ affect charge compensation and conductivity. Because ions penetrate the polymer bulk, analytes can strongly influence doping, giving OECTs exquisite sensitivity. In contrast, conventional organic semiconductors interact only at the film surface, leading to limited ion sensitivity, low transconductance, and the need for higher operating voltages. Their hydrophobic nature also reduces stability in aqueous environments, though they respond faster since they do not swell during measurement. Overall, OMIECs provide superior performance for continuous aqueous monitoring due to their bulk ion–electron coupling and stable operation in water [173,182–184].

### Recognition elements

FETs, chemiresistors, and working electrodes in electrochemical setups are not inherently selective. The selectivity can be built into the film or a separate material layered atop the film. In this section we discuss the different strategies that can be applied to make the film selective to specific targets: Introducing defects to the film, doping the film with nanoparticles, functionalizing the surface of the film with organic molecules or polymers, synthesizing nanocomposites of two or more materials, using biomaterials as sensing element, or coating the film with ISMs are different ways to create the recognition element in electrical and electrochemical sensing devices for ion detection.

#### Engineered structural nanoscale defect-rich films

Engineered structural nanoscale defects, such as atomic vacancies, grain boundaries, adatoms, or substitution sites, can serve as highly effective sensing sites in electronic and electrochemical ion sensors. In two-dimensional materials, for example, the creation of S-vacancies in MoS_2_ or point defects in graphene markedly enhance the chemical reactivity of the surface, allowing water-dissolved ions to adsorb or bind more strongly, thereby modulating the channel conductance or surface potential [159–160,185]. These defect sites often induce localized states in the bandgap or trap states that strongly couple to ionic charge, improving sensitivity and lowering detection limits in ion monitoring. Moreover, defects may enable preferential binding of specific metal ions; thereby, they can be engineered to tune sensing selectivity [185–186]. However, care must be taken because such defects can also degrade carrier mobility and increase noise or drift (especially in water where fouling or oxidation may occur); thus, balancing defect introduction and channel integrity is critical.

Defects can be introduced to the thin film with several techniques including electron beam or ion irradiation, plasma, ozone or chemical treatments, substitutional doping and ion implantation. These methods enable precise control over defect types and distributions, allowing researchers to tailor the electronic, and optoelectronic properties of thin films for advanced applications [186]. For instance, a MoS_2_-based electrochemical sensor was developed by creating nanoscale incisions and sulfur vacancies on MoS_2_ films by swift heavy ion (SHI) irradiation. SHI irradiation produces molybdenum-rich edges and removes sulfur atoms from the basal planes of MoS_2_, creating active sites for catalytic reactions to detect hydrogen ions (H^+^) in water. The irradiated MoS_2_ samples showed improved current densities and lower onset values for electrochemical reactions (i.e., lower LOD) [187]. Another study demonstrated that graphene’s pH sensitivity can be tuned by controlling its defect density. Different levels of defects (such as vacancies, dopants, functional groups, and sp^3^-hybridized regions) were introduced by sonication and chemical treatments. At high defect densities, the sensing response is dominated by the protonation and deprotonation of pH-sensitive functional groups (e.g., carboxy, hydroxy, and amino groups) on the graphene surface. These groups act as active sites for acid–base interactions, modulating graphene’s conductivity. The sensitivity of the chemiresistive graphene-based pH sensor has been shown to increase with higher defect density [188]. In summary, engineered structural defects provide a route to tailor-made high-sensitivity sensing sites in both electrochemical and electrical sensor formats.

#### Nanoparticle-doped films

**Figure 5:**
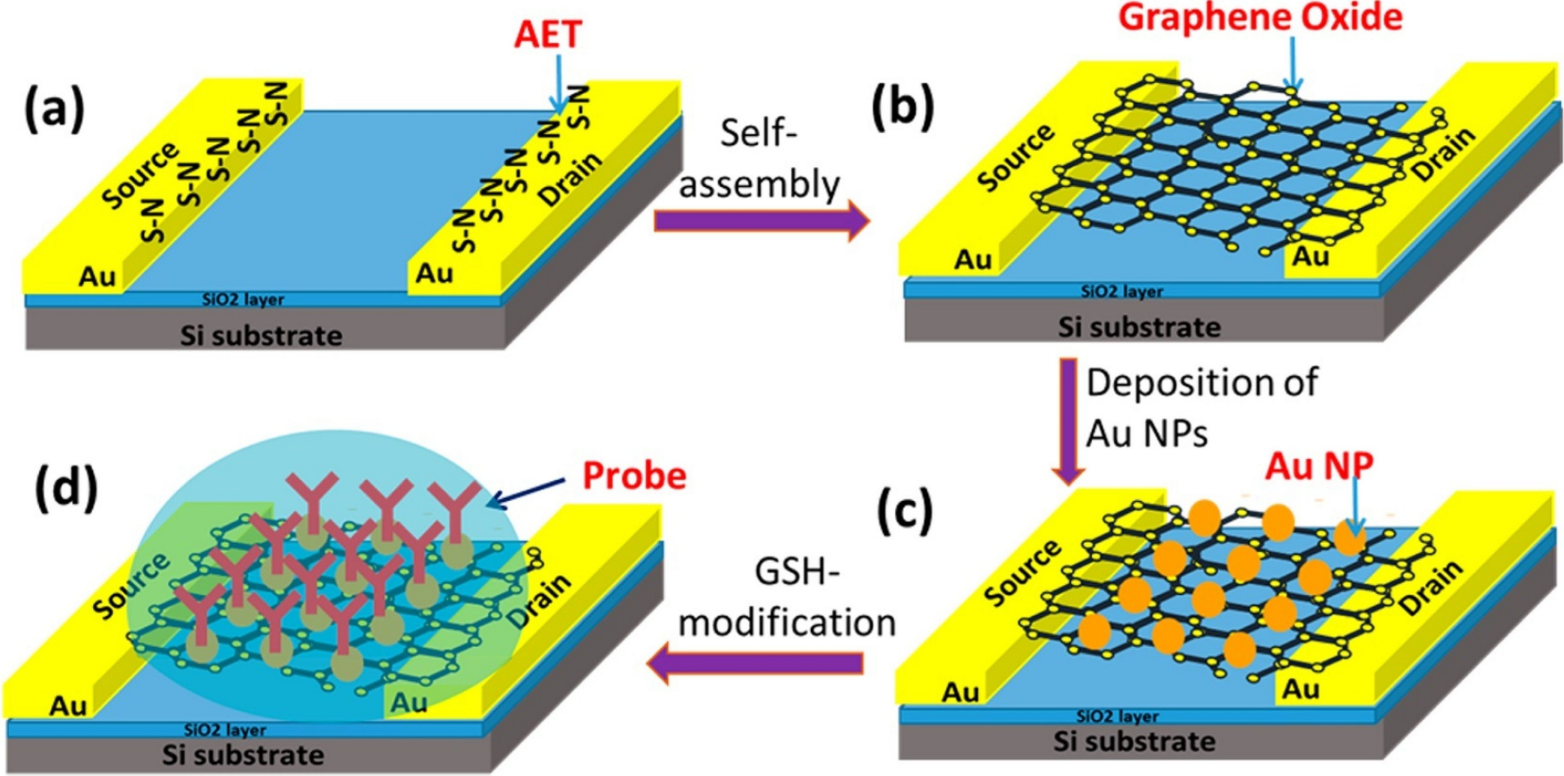
Schematic diagram of the rGO/GSH-Au NP hybrid sensor fabrication process. (a) A layer of AET coating on the bare interdigitated electrode surface. (b) Self-assembly of GO monolayer sheets on the AET-modified electrodes, which is followed by the thermal reduction of GO to rGO. (c) The assembly of Au NPs onto the rGO film. (d) GSH-modification of Au NPs on the rGO sheet surface to form specific recognition groups to detect Pb^2+^. Figure 5 was reprinted with permission from [191]. Copyright 2014 American Chemical Society. This content is not subject to CC BY 4.0.

Incorporating nanoparticles as dopants into sensor channel materials offers a powerful strategy to enhance sensing capability by introducing high‐activity sites and boosting signal transduction. When nanoparticles (NPs) such as Au, Ag, Pt are embedded within or on the surface of a channel material, they act as ion‐capture and catalytic centers, improving the local binding affinity for target ions, facilitating faster kinetics of ion–electron coupling, increasing the density of active sites, and lowering baseline resistance [189–190]. As an example, in a study done by Zhou et al., Au NPs play a central role in enhancing the electronic and sensing performance of the rGO-based sensor through their distinct electronic characteristics and chemical functionality (Figure 5). The higher work function of Au NPs (5.1–5.47 eV) relative to rGO (4.4–4.65 eV) drives electron transfer from rGO to the Au NPs, thereby increasing the hole concentration within the rGO channel and improving its electrical conductivity. Although the incorporation of Au NPs introduces scattering sites that can locally hinder charge-carrier mobility, the dominant effect of charge transfer leads to an overall increase in current flow through the device. Beyond their electronic influence, Au NPs can serve as platform for further functionalization due to their strong affinity for thiolated molecules. The immobilization of glutathione (GSH) onto the Au NP surface enables selective coordination of Pb^2+^ ions, thereby significantly enhancing both the sensitivity and specificity of the sensing interface [191].

Therefore, nanoparticle doping enhances sensor performance by amplifying signals via high‐surface‐area nanostructures, enabling tailored selectivity through functionalized NP dopants. However, potential issues include NP agglomeration or leaching during extended aqueous exposure, baseline noise or drift due to complex NP–matrix interfaces, and added fabrication complexity or cost. Thus, critical design priorities involve stabilizing NPs, controlling their distribution and size for reproducible sensor responses, and ensuring long‐term durability in real water matrices [29,192].

#### Molecularly functionalized films

Molecules such as ion‐chelating ligands, self-assembled monolayers, or crown ethers functionalized onto the electrode or transistor channel surface can create discrete recognition sites and significantly enhance selectivity and sensitivity of ionic sensors [29,193–194]. Such molecular layers also serve to position the ionic binding event within the electrical double layer (EDL) zone adjacent to the transducer surface, maximizing coupling and signal transduction. Studies of EDL accumulation show how ion binding at functionalized interfacial sites amplifies charge modulation in solution‐gated devices [195]. Moreover, surface functionalization can help mitigate non‐specific adsorption and fouling in complex water matrices by introducing hydrophilic groups that serve an antifouling function. Indeed, hydrophilic groups bind water strongly and create a hydration shell. This hydrated layer minimizes hydrophobic interactions, reduces van der Waals attraction, and increases steric repulsion. Therefore, biofouling and nonspecific adsorption can be suppressed by surface functionalization [29].

Molecules can be bound covalently or noncovalently onto the active layer. As an example of covalent functionalization, Kaur et al. fabricated an electrode by electro-copolymerizing PEDOT and 2-(anthracen-9-yl)benzo[*d*]thiazole (AA-BTZ) and then drop-casting Au NPs to create receptor sites for Hg^2+^ ions. The sulfur atoms in the benzothiazole moiety of AA-BTZ provide a soft base environment that complements the soft acid nature of Hg^2+^ ions, following the hard–soft acid–base (HSAB) principle. AuNPs further enhanced selectivity and sensitivity of the sensor due to their strong binding affinity with Hg^2+^ ions, resulting in a low detection limit of 0.60 nM [196].

Dalmieda et al. reported a chemiresistive Ag^+^ sensor based on FLG that was noncovalently functionalized with bathocuproine via π–π stacking [99]. It is a noncovalent strategy that preserves the intrinsic electronic properties of graphene while introducing controlled n-doping through electron donation from the adsorbed molecules. The sensing mechanism is governed by the selective formation of a silver–bathocuproine complex upon exposure to Ag^+^ ions, which alters the electronic structure of the ligand and creates electron-trapping sites at the graphene interface. This induces charge transfer from the FLG surface to the complex, increasing the hole density within the graphene film and producing a measurable decrease in resistance proportional to the Ag^+^ concentration. This strategy of sensing is limited to ions that are willing to form planar complexes, such as Ag^+^ and Cu^2+^(/Cu^+^) [197]. The chelation process is fully reversible since protonation of the bathocuproine imine groups under mildly acidic conditions (pH 3) disrupts complex formation and restores the baseline conductance, enabling sensor regeneration. Through this charge-transfer-driven modulation of film resistance, the platform achieves sensitive and selective Ag^+^ detection across a range of 3 ppb to 1 ppm in aqueous media [99].

#### Polymer-functionalized films

Engineered porous materials such as metal–organic frameworks (MOFs), covalent organic frameworks (COFs), and IIPs can directly incorporate sensing sites for ion detection in water via electrochemical or electrical devices. These frameworks combine high surface area, tunable pore structures, and specific binding chemistries that enhance ion uptake and transduction.

**Metal–organic frameworks.** MOFs are crystalline materials with highly ordered pores and channels. This uniform network structure provides controlled pathways for electron and ion transport; so, ion diffusion pathways are predictable. Ions travel through well-defined pores rather than random spaces, which results in faster diffusion, reproducible sensor responses, and better signal stability. Also, pore size and chemistry of MOFs can be precisely tuned. They can be hydrophilic or hydrophobic pores with selective coordinative sites, specific electrostatic charges, specific pore diameters. Moreover, MOFs have large specific surface areas (1000–7000 m^2^/g) with many active sites for adsorbing analytes or creating redox-active centers that are accessible, resulting in signal amplification and high sensitivity. MOFs can also be further functionalized, and due to their large surface area, a higher loading of catalytic or recognition molecules is possible. Furthermore, many MOFs contain metal nodes that are not fully coordinated. These unsaturated metal sites act like Lewis acid sites and bind analytes, act as redox-active centers, or catalyze electrochemical reactions [198–199]. Selvaraj et al. reported a UiO-67–modified electrode for cadmium detection using differential pulse voltammetry. The highly mesoporous UiO-67 structure (998 m^2^/g specific surface area, 0.70 cm^3^/g specific pore volume) efficiently adsorbs and concentrates Cd^2+^ ions from solution. This accumulation enhances the interfacial electron transfer kinetics, evidenced by a marked decrease in redox peak separation compared with the bare electrode, enabling efficient electrooxidation of Cd^2+^. The natural affinity of UiO-67 for cadmium ions, adsorption capability, electrostatic enrichment, and electrocatalytic activity allow UiO-67 to function as a sensitive (with a LOD of 1.43 nM/µA) and low-cost platform for trace-level Cd^2+^ monitoring in aqueous media [200].

Most MOFs are made of electrically insulating organic linkers and metal clusters that are not in direct electronic communication. As a result, typical MOFs have very low conductivities of around 10^−10^–10^−15^ S/cm. Nevertheless, there have been efforts to specifically develop conductive MOFs, enabling their use in FET sensors. Shen et al. synthesized a conductive Ni_3_(HITP)_2_ membrane via a one-step hydrothermal process, subsequently functionalizing it with glutaraldehyde and single-stranded DNA probes and measuring Hg^2+^ selectively through thymine–Hg^2+^–thymine coordination within the SG-FET configuration. The Ni_3_(HITP)_2_ membrane was structurally optimized by regulating the ammonia–water ratio to improve its carrier mobility and strengthen its transduction efficiency. This combination of conductive MOF architecture and specific nucleic-acid-based binding afforded a highly sensitive and selective sensor capable of detecting Hg^2+^ over a wide dynamic range from 10 pM to 100 nM [201]. Several challenges remain in the use of MOFs, such as their instability in aqueous environments. This is particularly true for carboxylate-based MOFs, which undergo hydrolysis because metal–ligand bonds such as Zn–O and Cu–O are not resistant to water or humidity. Another limitation is their poor adhesion to electrode surfaces, as MOFs are inherently crystalline powders with limited chemical affinity, covalent bonding, or mechanical compatibility with substrates. As a result, particles often detach during rinsing, cycling, or sonication. These drawbacks can be mitigated by designing MOF-based composites that enhance surface adhesion, water stability, and electrical conductivity [198–199].

**Covalent organic frameworks.** COFs likewise offer robust, crystalline, covalently bonded porous networks that permit fast mass transport of water and ions as well as functionalization of binding sites [202]. Unlike MOFs, COFs bind through organic donor groups, not metal clusters. Ion sensing in COFs operates through four main mechanisms: (1) coordination binding, where donor atoms in the framework form bonds or strong interactions with incoming ions, guided by HSAB principles and geometry matching; (2) π-interaction and charge transfer, in which ions interact with the extended π-conjugated planes, leading to electron redistribution, charge transfer, and changes in conductivity; (3) size-selective pore sieving, as the rigid, periodic pores discriminate ions based on size, shape, and diffusion pathways; and (4) electrostatic attraction or repulsion, where charged pores enhance selectivity by drawing in or excluding specific ions. COFs are suitable for sensing due to their fast ion diffusion through ordered pores and high density of accessible binding sites (a large specific surface area of 1000–3000 m^2^/g). In contrast to the metal–ligand bonds in MOFs, COFs rely on organic covalent bonds, which can be highly water-stable if chosen appropriately. At the molecular level, COFs can be designed with selective chelators, redox-responsive groups, proton-binding moieties, and ion-exchange functionalities [202].

Hou et al. developed a flexible electrochemical sensor for ultratrace mercury detection that utilizes a COF-based AuNPs-modified carbon fiber cloth to achieve highly efficient signal transduction. The electrode was subsequently functionalized with methylene blue (MB) and rDNA to selectively interact with Hg^2+^ ions. In this scenario Hg^2+^ acts as a specific bivalent linker between thymidine bases on the rDNA probe, thus shifting the electroactivity of MB. The COF/Au/MB/rDNA probe architecture significantly enhances sensitivity through the high surface area and biocompatibility of the COF, efficient electron transfer pathways provided by Au NPs, and the MB signaling moiety. This synergistic design enables a broad linear detection range from 0.1 pM to 100 nM and a very low detection limit of 45.2 fM. The proposed sensor also demonstrates good stability (given the use of rDNA), reproducibility, and practical applicability, achieving high recovery rates in diverse real water samples, including tap, lake, drinking, and seawater [203]. In another study, Wang et al. developed a screen-printed COFDATA-TP-based electrochemical sensor for multiplex heavy-metal detection using the ASV method. The COFDATA-TP framework contains abundant carboxyl (–COOH) and secondary amino (–NH) functional groups, which serve as high-affinity binding sites, enabling efficient enrichment of Hg^2+^, Cu^2+^, Pb^2+^, and Cd^2+^ from solution and directing them to the electrode interface. The π-conjugated backbone of COFDATA-TP enhances electrical conductivity, while its ordered porous architecture promotes mass transport and facilitates rapid ion diffusion, collectively improving signal strength and sensitivity. The detection limits of the sensor to detect Hg^2+^, Cu^2+^, Pb^2+^, and Cd^2+^ are calculated to be 2.80, 5.01, 1.83, and 2.91 nM, respectively [204]. Similar to MOFs, COFs also tend to suffer from low intrinsic conductivity. They are often electrically insulating unless they are doped, prepared as composites with carbon materials or made with fully π-conjugated linkers. Also, their stability depends on linkage chemistry, for example imine COFs may undergo hydrolysis but ketoenamine COFs are more stable [202]. They may also suffer from weak adhesion to surfaces of substrates. Overall, COFs represent a promising platform for ion sensing, though advances in conductivity, stability, and surface adhesion remain essential for their widespread application.

**Ion-imprinted polymers.** IIPs are synthesized to create “molecular memory” cavities that match a target ion in size and coordination environment, enabling highly selective recognition, even in complex water matrices, with demonstrated nanomolar detection limits [205–206]. IIPs are highly selective because binding pockets are pre-organized around divalent or monovalent ions, they can discriminate different geometries and coordination numbers (octahedral (e.g., Co^2+^), tetrahedral (e.g., Zn^2+^), linear (e.g., Ag^+^)). Moreover, they do donor-atom matching based on HSAB theory (soft S donors to Hg^2+^ and Ag^+^, hard O donors to Ca^2+^and Mg^2+^). In addition, polymer cavities reject ions that are too large or ions with mismatched hydration radius or incorrect geometry due to their steric constraints. During sensing, ions migrate through the aqueous medium into the polymer’s surface pores where electrostatic forces guide them toward donor atoms. Within these cavities, the ions coordinate with donor sites to form stable complexes. This interaction induces subtle conformational adjustments in the polymer, such as localized movements of chain segments or side groups, that help secure the ion within the binding pocket. As a result, the sensor generates a measurable transduction signal. The IIP-based sensing is fast (because cavities are pre-formed), highly selective (due to geometry and donor atom matching), and stable (chelation-like binding) [150].

As an example, Zhang et al. synthesized Ni(II)-imprinted polymers on a magnetic MWCNT substrate using a surface imprinting technique and selectively detected Ni^2+^ ions with a LOD of 0.27 µg/L. These imprinting cavities are tailored in size, geometry, and charge distribution to match Ni^2+^, which enables selective adsorption. When an applied potential drives Ni^2+^ ions into the binding sites, they coordinate with functional monomer groups through electrostatic and ligand–metal interactions. Incorporation of Fe_3_O_4_@MWCNTs into the electrode matrix substantially enhances electron-transfer kinetics and increases the effective electroactive surface area approximately fourteen-fold compared with a bare electrode, yielding a markedly amplified electrochemical response [207].

**Figure 6:**
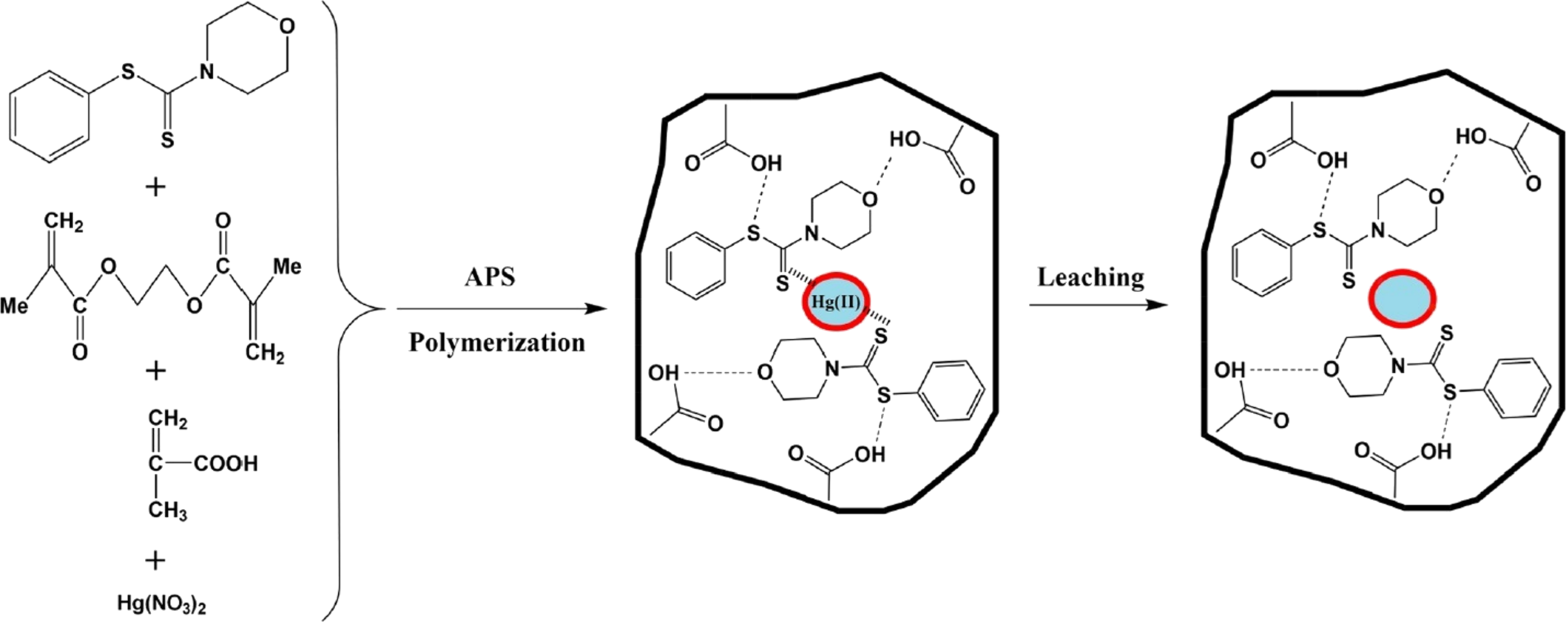
Schematic representation of the fabrication of Hg(II)-IIP. Figure 6 was reproduced from [205] (© 2024 E. Shamsabadi et al., published by Springer Nature, distributed under the terms of the Creative Commons Attribution-NonCommercial-NoDerivatives 4.0 International License, https://creativecommons.org/licenses/by-nc-nd/4.0/). This content is not subject to CC BY 4.0.

Shamsabadi et al. developed a novel IIP–modified carbon paste electrode through a simple bulk polymerisation process, which was able to selectively measure Hg^2+^ ions with a LOD of 0.2 nM (Figure 6). The sensing mechanism proceeds through sequential preconcentration, reduction, and stripping steps. During the open-circuit preconcentration stage, Hg^2+^ ions in solution selectively bind to morpholine-4-carbodithioic acid phenyl ester (MCP) sites within the imprinted polymer matrix, forming Hg^2+^–MCP complexes that accumulate at the electrode surface. Upon applying a potential of −0.5 V, the preconcentrated Hg^2+^ is electrochemically reduced to elemental mercury (Hg^0^), regenerating the MCP ligand in the process. In the subsequent differential pulse anodic stripping voltammetry (DPASV) step, the deposited Hg^0^ is reoxidized to Hg^2+^, producing a well-defined anodic peak that serves as the quantitative analytical signal [205].

In summary, MOFs, COFs, and IIPs can act as pre-concentration and binding layers at the interface of a sensor such that ion binding causes modulation of surface potential, ionic flux, or channel conductivity. Despite their promise, several challenges persist: MOFs and COFs often face stability issues in aqueous environments, IIPs require careful management of fouling and regeneration, and seamless integration with electronic transducers must be achieved without introducing drift or baseline noise. Even so, their porous architectures, rapid response, and very high selectivity and sensitivity establish these materials as leading candidates for robust, real-time ion sensing in water.

#### Nanocomposite films

Engineered nanocomposites, hybrid materials composed of two or more nanoscale phases (e.g., carbon nanostructures + metal/metal-oxide nanoparticles or conjugated polymers) offer powerful advantages as ion sensing sites. By combining the high surface area of 2D materials and nanotubes (e.g., graphene, MXenes, CNTs) with functional modifiers (ionophore polymers, metal-oxide nanostructures), these composites can provide enhanced ion adsorption/binding, accelerated charge transfer, and stable aqueous operation [107,208]. When the density of active sites increases, the sensitivity of the sensor improves. Moreover, enhanced electron and ion transport pathways lead to faster kinetics, and the underlying conductive framework prevents agglomeration or leaching, which results in more stable devices [107]. Zhang et al. developed a one-step fabricated electrochemical sensor for highly selective Pb^2+^ detection using an integrated TCPP-MWCNTs@Fe_3_O_4_ nanocomposite probe, in which the sensing mechanism is governed by the selective chelation of lead ions by *meso*-tetra(4-carboxyphenyl)porphyrin (TCPP). The tetrapyrrolic cavity of TCPP provides electron-rich nitrogen coordination sites that selectively accommodate Pb^2+^, forming a stable chelate that enables highly discriminative recognition over competing metal ions. Aminated MWCNTs serve as a conductive scaffold for covalent immobilization of TCPP through amidation, creating a high-surface-area interface that promotes Pb^2+^ adsorption while facilitating efficient electron transport during electrochemical transduction. The incorporation of Fe_3_O_4_ nanoparticles imparts magnetic responsiveness, allowing for rapid magnetic separation and direct assembly of the nanocomposite onto a magnetic glassy carbon electrode, thereby integrating pre-concentration, probe immobilization, and detection into a single streamlined step. Together, selective porphyrin–Pb^2+^ chelation, enhanced conductivity of the MWCNT network, and magnetic fixation of the sensing interface result in a stable, regenerable, and highly sensitive platform capable of detecting Pb^2+^ with a LOD of 0.067 ppt [209].

**Figure 7:**
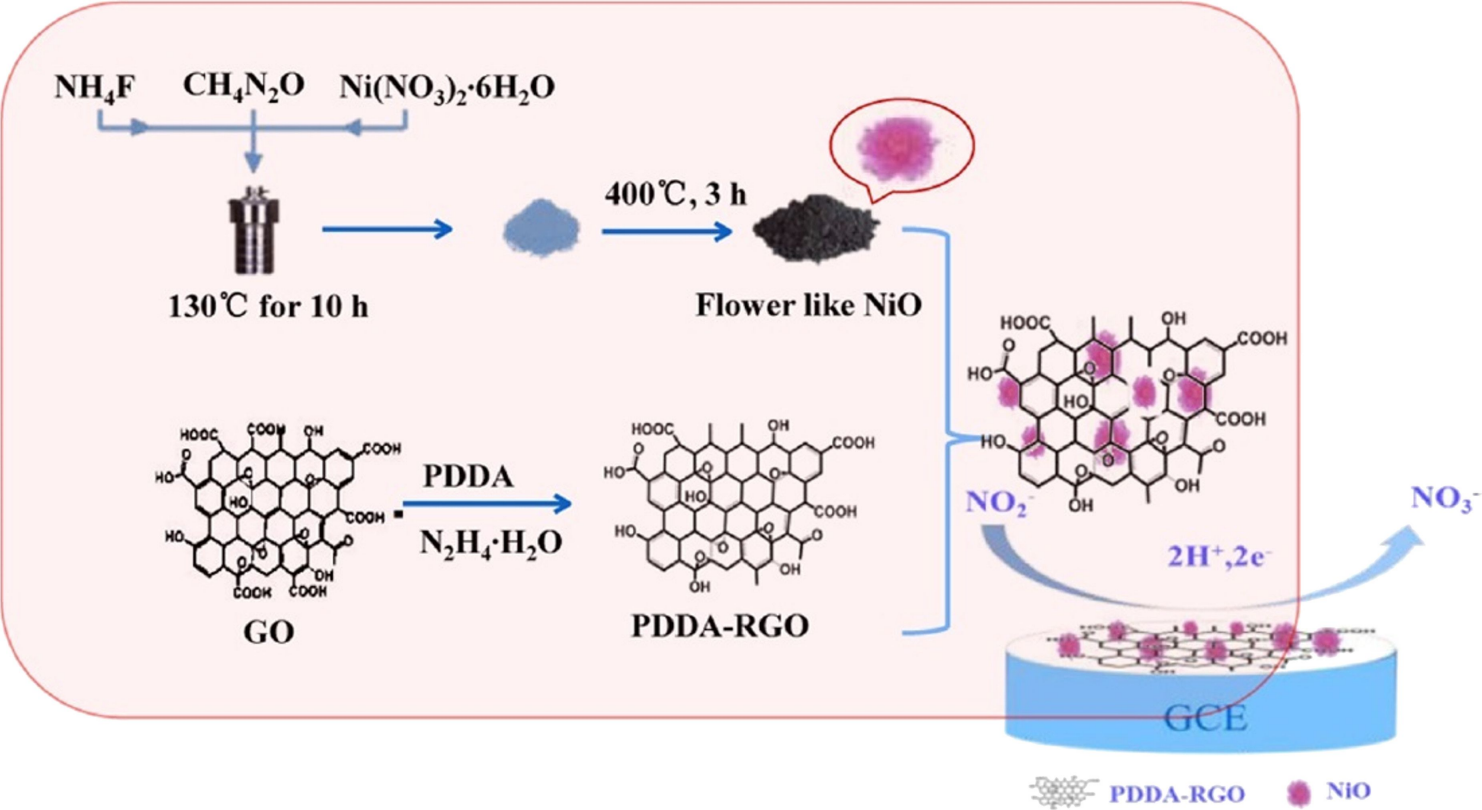
Scheme of the preparation route for the NiO/PDDA-RGO composite and its electrochemical application for nitrite detection. Figure 7 was reprinted with permission from [211]. Copyright 2022 American Chemical Society. This content is not subject to CC BY 4.0.

Sharma et al. reported a highly sensitive platform for real-time mercury ion detection based on an Ag nanowire–MoS_2_ nanocomposite integrated onto the gate region of an AlGaN/GaN HEMT. The sensing mechanism arises from the interaction of Hg^2+^ ions with the MoS_2_ surface within the nanocomposite, which modulates the gate potential and, consequently, the drain-to-source current (*I*_DS_) of the HEMT. At low Hg^2+^ concentrations, strong chemisorption and the formation of Hg–S complexes dominate, increasing the gate potential and producing a corresponding rise in *I*_DS_. As the concentration increases and MoS_2_ binding sites become saturated, additional Hg^2+^ uptake occurs primarily through electrostatic attraction, leading to a reduction in surface potential and a subsequent decrease in *I*_DS_. This dual-stage response enables highly discriminative electrical transduction. Combining AgNWs with MoS_2_ results in a nanocomposite with superior electron transport properties and more adsorption sites, enabling the sensor to achieve a sensitivity of 1.604 mA/ppb and a very low detection limit of 20 ppt, which is the best reported for AlGaN/GaN HEMT-based sensors for Hg^2+^ detection [210]. Another group of researchers reported a 3D flower-like nickel oxide-decorated poly(diallyldimethylammonium chloride) (PDDA)-functionalized rGO nanocomposite (Figure 7). The NiO/PDDA-rGO electrochemical sensor detects nitrite through a diffusion-controlled catalytic oxidation process facilitated by the synergistic properties of the nanocomposite. Nitrite ions are first adsorbed onto the NiO/PDDA-rGO surface, forming a transient NiO/PDDA-rGO–NO_2_^−^ complex through electrostatic interactions. This interfacial complex undergoes electron transfer, yielding nitrogen dioxide (NO_2_), which subsequently disproportionates to generate additional nitrite (NO_2_^−^) and nitrate (NO_3_^−^). The newly formed nitrite is further electrooxidized to nitrate as the final product, producing the characteristic oxidation current used for quantitative detection. The high sensitivity of the sensor arises from the combination of flower-like NiO nanostructures that offer abundant catalytic sites and the PDDA-functionalized rGO matrix that enhances charge transport and adsorption, collectively enabling an operating range of 5 μM−8 mM and a detection limit of 0.98 μM [211]. Despite the benefits of nanocomposites, some new challenges arise such as achieving robust dispersion and a uniform interface between components (to avoid baseline drift or noise) or fabricating reproducible and scalable sensors, which require careful consideration in sensor development [208].

#### Biomolecule-functionalized films

Biomolecules such as enzymes, DNA, aptamers, antibodies, and DNAzymes can serve as precise recognition elements for sensing applications. Aptamers or single‐strand nucleic acids selected via systematic ligand evolution by exponential enrichment can bind specific metal ions with high affinity and be integrated into electrical and electrochemical sensors [212–213]. DNAzymes (catalytic nucleic acids) can change their conformation or activity in response to specific ions, generating a measurable electrical signal when immobilized on an electrode [214]. Enzymes and antibodies provide complementary functionality. Enzymes can catalyze ion‐related reactions (for instance, urease producing NH_4_^+^ from urea, leading to pH changes), while antibodies bind specifically to ion complexes (not free ions because free metal ions are very small and hard for antibodies to recognize) and enable high‐selectivity immunosensors [215]. Zhang et al. designed an ultrasensitive electrochemical aptasensor for Pb^2+^ detection. Ti_3_C_2_T*_x_* as a substrate has outstanding metallic conductivity for efficient electron transfer, good biocompatibility, and a high surface area with abundant reactive surface sites to facilitate stable immobilization of biomolecular probes. When combined with polypyrrole and gold nanoparticles, Ti_3_C_2_T*_x_* also exhibits improved oxidative stability, resulting in a robust platform for constructing high-performance aptasensing interfaces. After preparation of the Ti_3_C_2_T*_x_*/PPy/AuNPs nanocomposite, the capture probe immobilized on the electrode hybridizes with the lead-specific aptamer probe to form a duplex DNA structure that anchors MB as the electrochemical indicator. Upon exposure to Pb^2+^, the aptamer undergoes a selective conformational switch from the double helix to a Pb^2+^-stabilized G-quadruplex, triggering the release of MB from the electrode surface and producing a measurable decrease in current proportional to the Pb^2+^ concentration. Collectively, the Pb^2+^-responsive G-quadruplex transition and the MXene-based conductive architecture enabled a highly sensitive measurement of Pb^2+^ ions in a range from 5 × 10^−14^ to 1 × 10^−8^ M with a low LOD of 1 × 10^−14^ M [216].

**Figure 8:**
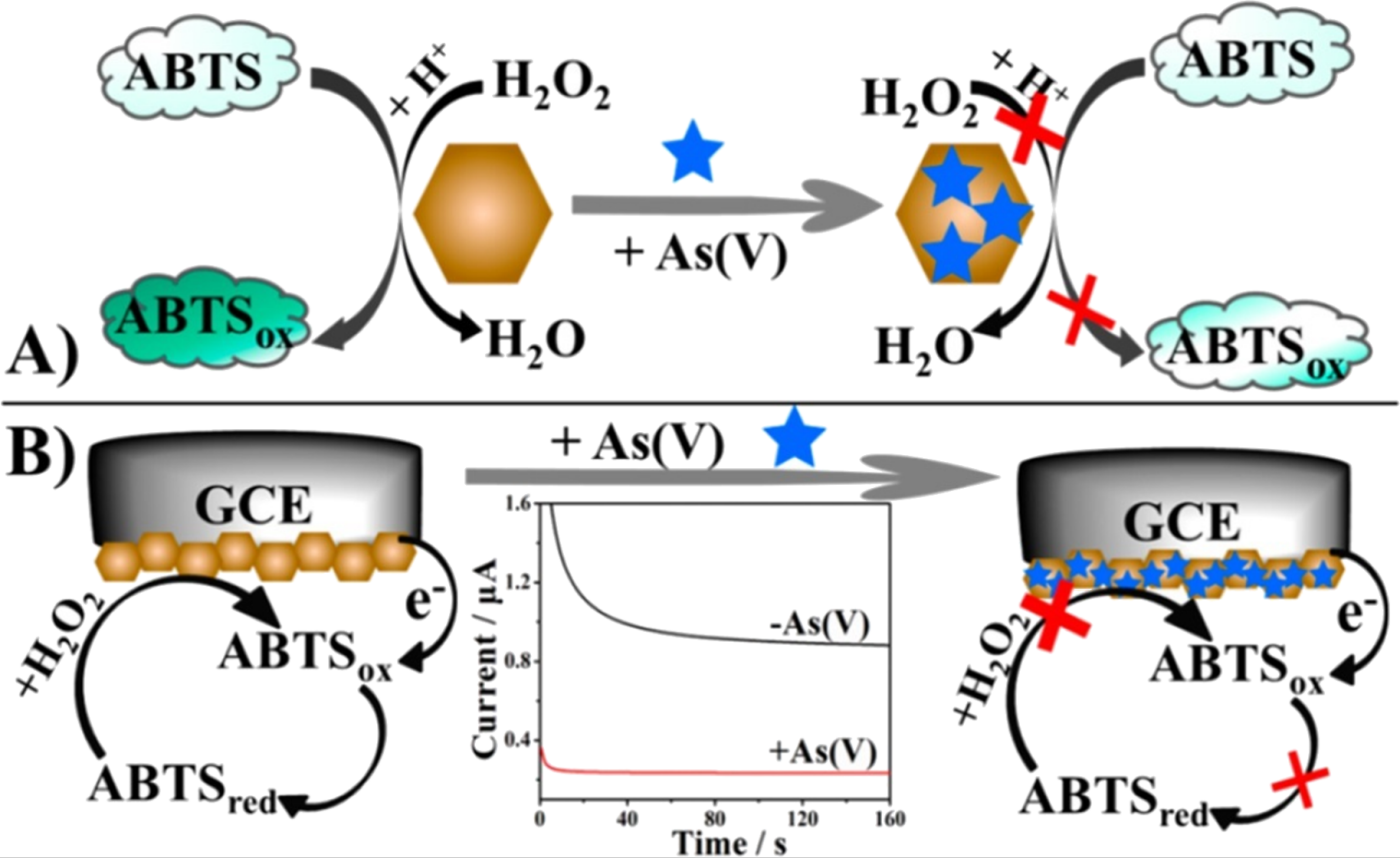
Schematic illustration of the dual-mode assay of arsenate based on CoOOH nanoflakes with peroxidase-like activity: (A) Illustration of the colorimetric method of CoOOH nanoflakes as probe for arsenate detection; (B) Illustration of the electrochemical strategy of CoOOH nanoflakes modified glassy carbon electrode for arsenate detection. Figure 8 was reprinted with permission from [218]. Copyright 2019 American Chemical Society. This content is not subject to CC BY 4.0.

Nanozymes are synthetic nanomaterials that mimic the catalytic activity of natural enzymes. They offer relatively high stability, low cost, and tunable surface properties, enabling enzyme-like functions without the fragility of biological catalysts [217]. Wen et al. utilized cobalt oxyhydroxide (CoOOH) nanoflakes as a nanozyme to detect arsenate ions (As^5+^) (Figure 8). The sensing mechanism relies on the strong electrostatic and surface-complexation interactions between As(V) and CoOOH, where the formation of As–O coordination complexes inhibits the intrinsic peroxidase-like activity of the nanozyme. This suppression reduces the catalytic oxidation of 2,2′-azino-bis(3-ethylbenzothiazoline-6-sulfonic acid) (ABTS) to ABTS_ox_, resulting in a measurable decrease in the electrochemical reduction current of ABTS_ox_ during transduction. The degree of signal inhibition correlates directly with As(V) concentration, enabling ultrasensitive detection with a limit of 56.1 ppt [218]. The advantages of biomaterials in ion sensing include excellent selectivity, potential for regeneration, compatibility with miniaturized electronics, and adaptability to real‐time, in‐line measurements in flowing water. However, challenges arise in terms of their long‐term stability in complex water matrices (biofouling, denaturation, drift), and the need to interface them effectively with solid‐state transducers (electrode functionalization, stable immobilization) to maintain signal accuracy. Proper integration of biomaterial recognition layers with electrical and electrochemical surfaces thus enables high‐performance ion sensing platforms for water quality monitoring [215].

#### Ion-selective membrane-coated films

Throughout the review, the prominent role of ISMs has been emphasized repeatedly. These membranes act as recognition layers, enabling the selective conversion of ion activity in the aqueous phase into measurable electrical signals. Typically, an ISM consists of a plasticized polymer matrix incorporating an appropriate ionophore, often supplemented with a lipophilic additive that enhances ionophore performance through electrostatic interactions, thereby improving ion selectivity. In ISEs, the membrane binds specifically to the target ion and generates a phase boundary potential, which is detected by a transducer. Within the field of SC-ISEs, extensive research has focused on optimizing ISM composition and refining membrane deposition techniques to create sensing platforms with high reproducibility and minimal signal drift [75–76,219]. A recent study by Yu et al. reported an all-solid-state potassium ISE. Potassium ions in solution are selectively complexed by the cyclo-dodecapeptide (CP12) ionophore embedded in the K^+^-selective carrier membrane, generating a transmembrane ion-activity gradient that produces a diffusion potential consistent with Nernstian behavior. This ionic potential is converted into a stable electronic signal by an underlying layer of egg-shell hollow carbon nanospheres, whose high capacitance facilitates rapid charge transfer while the hydrophobic carbon surface suppresses water-layer formation between the membrane and electrode substrate. Together, these features yield fast response kinetics, high sensitivity, and excellent potential stability, enabling reliable K^+^ detection over a range of concentration from 5.0 µM to 10 mM even in complex sample matrices [220].

Although ISMs are fundamental components of potentiometric ISEs, they are also employed, albeit less frequently, in amperometric, voltammetric, and FET-based sensors to improve selectivity, facilitate ion preconcentration, minimize interference, and protect surfaces. When positioned over a transistor channel, as in ChemFET configurations, ISMs establish ionic gating or ion-specific accumulation layers that modulate the channel’s surface potential. Unlike potentiometric ISEs, where the membrane generates a measurable phase boundary potential, detection in this geometry arises from changes in channel conductance governed by ion access to the semiconductor surface. Liu et al. developed an ISM-ISFET sensor for Cr^6+^detection. Under mildly acidic conditions, Cr^6+^ exists primarily as Cr_2_O_7_^2−^, which binds to quinaldine red ionophores in the membrane, triggering a proton-releasing reaction that alters the membrane-solution interfacial potential. This potential modulation directly affects the ISFET gate surface, producing a measurable change in drain current corresponding to Cr^6+^ concentration. To suppress background noise and eliminate signal drift from nonspecific ions or pH fluctuations, a differential sensing architecture is used in which ISFET pixels coated with the ISM are compared against uncoated reference pixels, and the resulting differential current (Δ*I*_D_) isolates the Cr^6+^-specific response. A microfluidic control system further stabilizes the pH and ensures precise solution mixing, enhancing signal accuracy and the overall reliability of Cr^6+^ quantification in complex aqueous samples [221].

**Figure 9:**
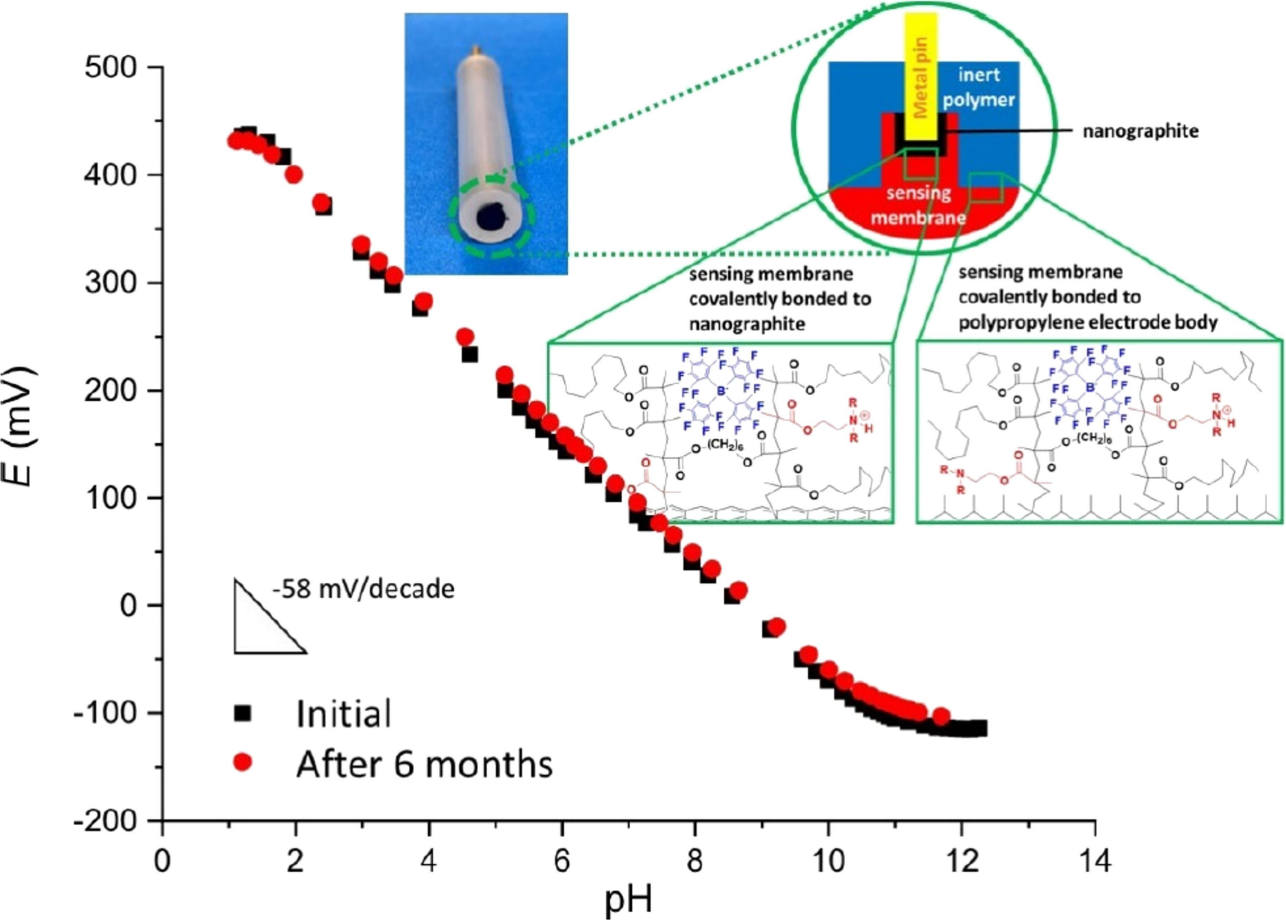
Initial EMF response (squares) and response after 6 months (circles) to pH for an ISE with a PDMA membrane (doped with ionophore and ionic sites) photografted onto a polypropylene-based electrode body and nanographite solid contact, relative to a free-flowing double-junction reference electrode. The pH was adjusted by the addition of 1.0 M HCl or 1.0 M NaOH to 10 mM sodium phosphate buffer solution (pH 7.1). The pH value shown on the *x*-axis was measured using a pH glass electrode. Figure 9 was reproduced from [219] (© 2023 K. R. Choi et al., *Angewandte Chemie, International Edition* published by Wiley-VCH GmbH, distributed under the terms of the Creative Commons Attribution-NonCommercial-NoDerivatives 4.0 International License, https://creativecommons.org/licenses/by-nc-nd/4.0/). This content is not subject to CC BY 4.0.

In potentiometric systems, ISM-coated electrodes form part of the electrochemical cell. In other sensing approaches, however, membranes may be less advantageous, as they can impede electron transfer, introduce diffusion barriers, or reduce catalytic efficiency. Moreover, incorporating membranes increases fabrication complexity. In many cases, selectivity can instead be achieved through direct surface modification strategies, such as self-assembled monolayers, chelating agents, polymers, or nanomaterials. Consequently, ISMs are most valuable when high selectivity is required, interfering ions are prevalent, ion preconcentration is beneficial, or surface fouling must be mitigated. Nonetheless, their use presents challenges, including membrane drift, ion interference, fouling, and ionophore leaching in complex water matrices, that demand robust membrane formulations and carefully designed transducers. Choi et al. recently introduced a durable ISE platform in which ionophore-doped poly(decyl methacrylate) (PDMA) sensing membranes are covalently anchored to both inert polymeric supports and conductive carbon contacts, overcoming longstanding issues of membrane delamination, water-layer formation, and signal drift common in conventional ISEs (Figure 9). Using a two-step photo-induced graft polymerization method, a robust chemical bonding between the sensing membrane and electrode substrates was achieved, thereby stabilizing the interfacial structure essential for reliable potentiometric transduction. The resulting electrodes exhibited excellent performance metrics, including highly reproducible standard potentials (electrode-E_0_ SD = 0.2 mV), minimal long-term drift (7 μV/h over 260 h), and full retention of sensing capability following harsh autoclave sterilization (121 °C, 2 atm). Furthermore, the devices maintained stable operation after six months of storage and exposure to elevated temperatures and organic solvents. Although demonstrated using pH-selective membranes, the approach is readily extendable to other target ions through appropriate ionophore incorporation, representing a significant advance toward robust, miniaturizable, and long-lived ISEs suitable for wearable, implantable, and industrial sensing environments [219].

### Sensor arrays

Sensor arrays are groups of multiple sensors arranged to simultaneously measure different ions or improve sensitivity and selectivity. Instead of using one sensor that is perfectly selective for a single ion, an array of sensors can be used in which each sensor has a different binding chemistry and responds differently to each ion in the sample. By analyzing all responses together, the array can identify or quantify ions with higher specificity. There are two types of arrays which are developed, namely, arrays of single-analyte sensors and cross-reactive arrays (electronic tongue).

#### Arrays of single-analyte sensors

These types of sensor arrays consist of multiple sensing units, each engineered to detect a single ionic species. The outputs from these sensors can be directly interpreted without reliance on advanced chemometric methods. Such arrays are typically constructed using ion-selective membranes or molecular recognition components such as ionophores, crown ethers, or IIPs. They enable the simultaneous measurement of several ions, with each channel functioning independently and providing orthogonal chemical selectivity. These arrays deliver high accuracy and allow for straightforward quantification, minimizing the need for computational correction or pattern recognition. Their reliability makes them particularly valuable in contexts requiring precise ion determination, including blood electrolyte analysis and water quality monitoring [222–223].

**Figure 10:**
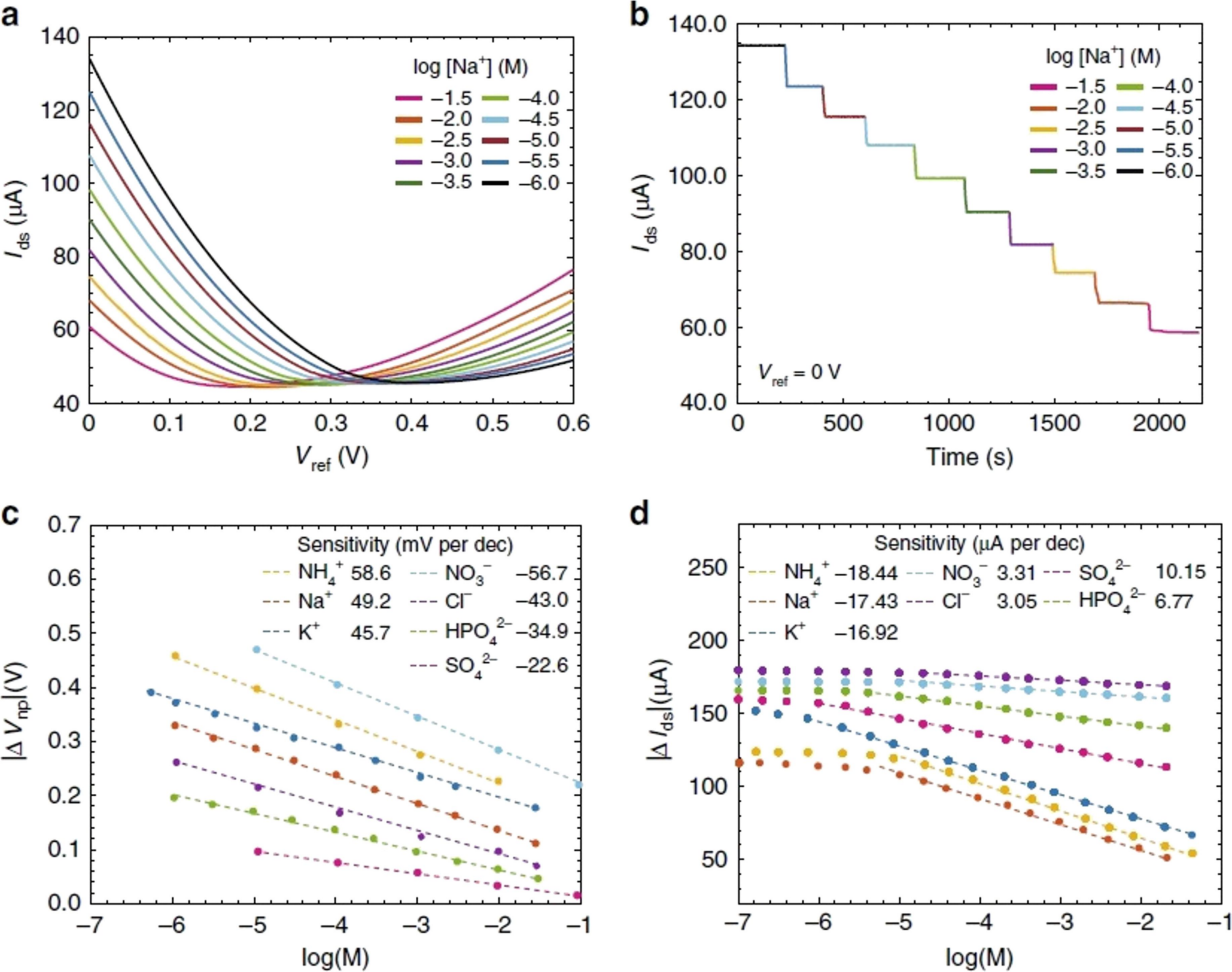
IS-FET sensitivities with respect to their target ions. a) The channel current, *I*_ds_, of an Na^+^ IS-FET versus *V*_ref_, for different Na^+^ molar concentrations. b) Continuous real-time measurement of *I*_ds_ for the same Na^+^ IS-FET when increasing Na^+^ molar concentrations by half decade steps while keeping *V*_ref_ = 0 V. c) The changes in *V*_np_ (neutrality point voltage, it is the gate voltage at which the channel has minimum conductance) for the different IS-FETs with respect to concentrations of their respective target ions, with a linear fit to extract their voltage sensitivities. d) The changes in *I*_ds_ for the different IS-FETs with respect to concentrations of their respective target ions, with a linear fit to extract their current sensitivities. Figure 10 was reproduced from [10] (© 2020 I. Fakih et al., published by Springer Nature, distributed under the terms of the Creative Commons Attribution 4.0 International License, https://creativecommons.org/licenses/by/4.0).

Fakih et al. reported a graphene-based IS-FET array capable of real-time, high-resolution, and simultaneous detection of multiple cations (K^+^, Na^+^, NH_4_^+^) and anions (NO_3_^−^, SO_4_^2−^, HPO_4_^2−^, Cl^−^) (Figure 10). The device was fabricated by growing a monolayer of graphene via CVD, which was then transferred onto a fused silica substrate. Source and drain contacts (Ti/Au) were deposited, after which the wafer was diced into individual chips, mounted on printed circuit boards, and encapsulated with epoxy to prevent direct contact with the electrolyte. ISMs tailored to specific ions were drop-cast onto the graphene surface to enable selective sensing. The array achieved a resolution of 2 × 10^−3^ log concentration units, with sensitivities ranging from 22.6 to 58.6 mV/decade. Detection limits were approximately 10^−5^ M for all analytes. Performance was validated in multi-ion solutions and in an aquarium environment over a three-week period. As expected, the IS-FETs exhibited some cross-sensitivity to non-target ions, necessitating calibration via the Nikolskii–Eisenman method, which compensates for interference by solving nonlinear equations to accurately determine ion concentrations in complex mixtures. Overall, this graphene IS-FET array combines high resolution, accuracy, and scalability for diverse real-world applications [10].

#### Cross-reactive arrays (electronic tongues)

In complex environments, selectivity is low, interfering ions distort readings, biofouling and drift affect single sensors, and multiple ions interact nonlinearly. An electronic tongue is a system consisting of a sensor array made of multiple partially selective sensors, a transduction system (such as potentiometric, voltammetric, FET, and chemiresistive sensors), and a pattern recognition engine (machine learning or statistical models). All the sensors in the array overlap in their responses, together creating a fingerprint for each ion. Selectivity then emerges from the pattern of responses, not from the individual sensors [222,224–225].

Machine learning algorithms can be employed to interpret how ions influence the response patterns of sensor arrays. The typical workflow begins with the collection of training data, where the array is exposed to known concentrations of ions. Each sensor generates a multidimensional signal (for example, a 12-sensor array yields a 12-channel response vector), which serves as the input for computational analysis. Machine learning methods include principal component analysis for dimensionality reduction, linear discriminant analysis for classification, support vector machines, random forests, neural networks, and regression approaches such as partial least squares or principal component regression. One of these methods is applied to extract discriminative patterns that differentiate individual ions. Through this process, the model learns a mapping between sensor response profiles and ion concentrations. During actual sensing, the array produces a new response pattern that the trained model can decode to predict which ions are present, their concentrations, and even resolve complex mixtures in heterogeneous matrices. Machine learning compensates for sensor drift, temperature variations, baseline offsets, overlapping sensitivity, and nonlinear sensor behavior. Machine learning models can update or self-calibrate. In other words, instead of requiring each sensor to behave perfectly, machine learning finds the statistically relevant features [224–225].

**Figure 11:**
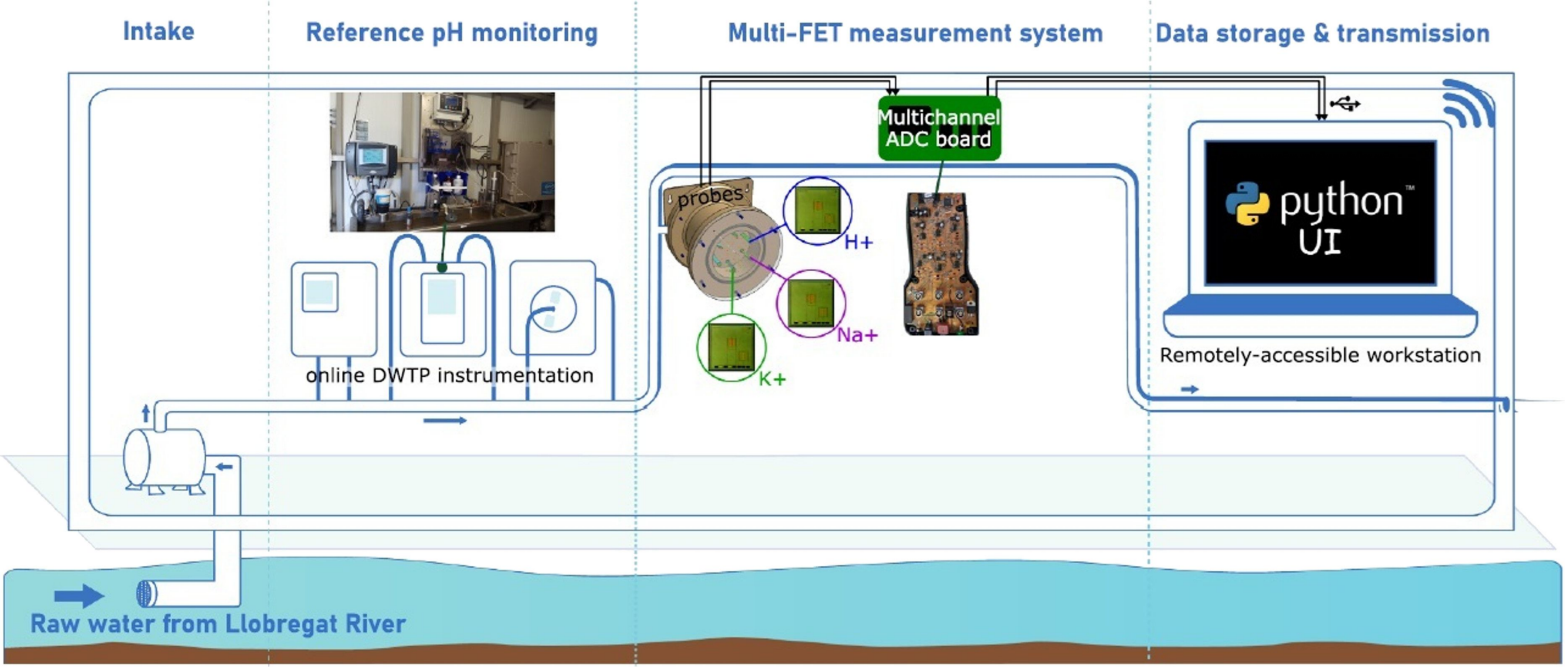
Experimental setup for continuously acquiring water quality data. The ISFET array was deployed in a custom probe connected to readout electronics fabricated in house at the Instituto de Microelectronica de Barcelona. A laptop received raw ISFET measurements via USB, for storage and wireless transmission. Figure 11 was reproduced from [226] (© 2021 J. M. Margarit-Taulé et al., published by Elsevier B.V., distributed under the terms of the Creative Commons Attribution-NonCommercial- NoDerivatives 4.0 International License, https://creativecommons.org/licenses/by-nc-nd/4.0/). This content is not subject to CC BY 4.0.

Margarit-Taulé et al. introduced an innovative strategy to mitigate common limitations of ISFET sensors in pH monitoring, including temporal drift, cross-sensitivity, and aging effects. Their approach integrates an array of ISFETs selective to H^+^ ions as well as interfering ions (Na^+^ and K^+^) with machine learning models (Figure 11). A range of ML algorithms, linear regression, support vector regression, multilayer perceptron, and deep neural networks (DNNs), were evaluated using sensor data collected over 90 days from a drinking water treatment facility. Among these, DNNs delivered the highest predictive accuracy. Incorporating signals from ISFETs targeting interfering ions significantly enhanced both stability and precision, with DNNs achieving a 73% reduction in root-mean-square error compared to conventional two-point calibration. This work highlights the promise of DNN-based sensor fusion in improving the robustness and operational lifespan of FET sensors for real-world applications such as water quality assessment. Moreover, the methodology can be extended to other potentiometric sensors for analyte detection in complex environments [226].

### Systematic evaluation of active materials for ion sensing in water

**Figure 12:**
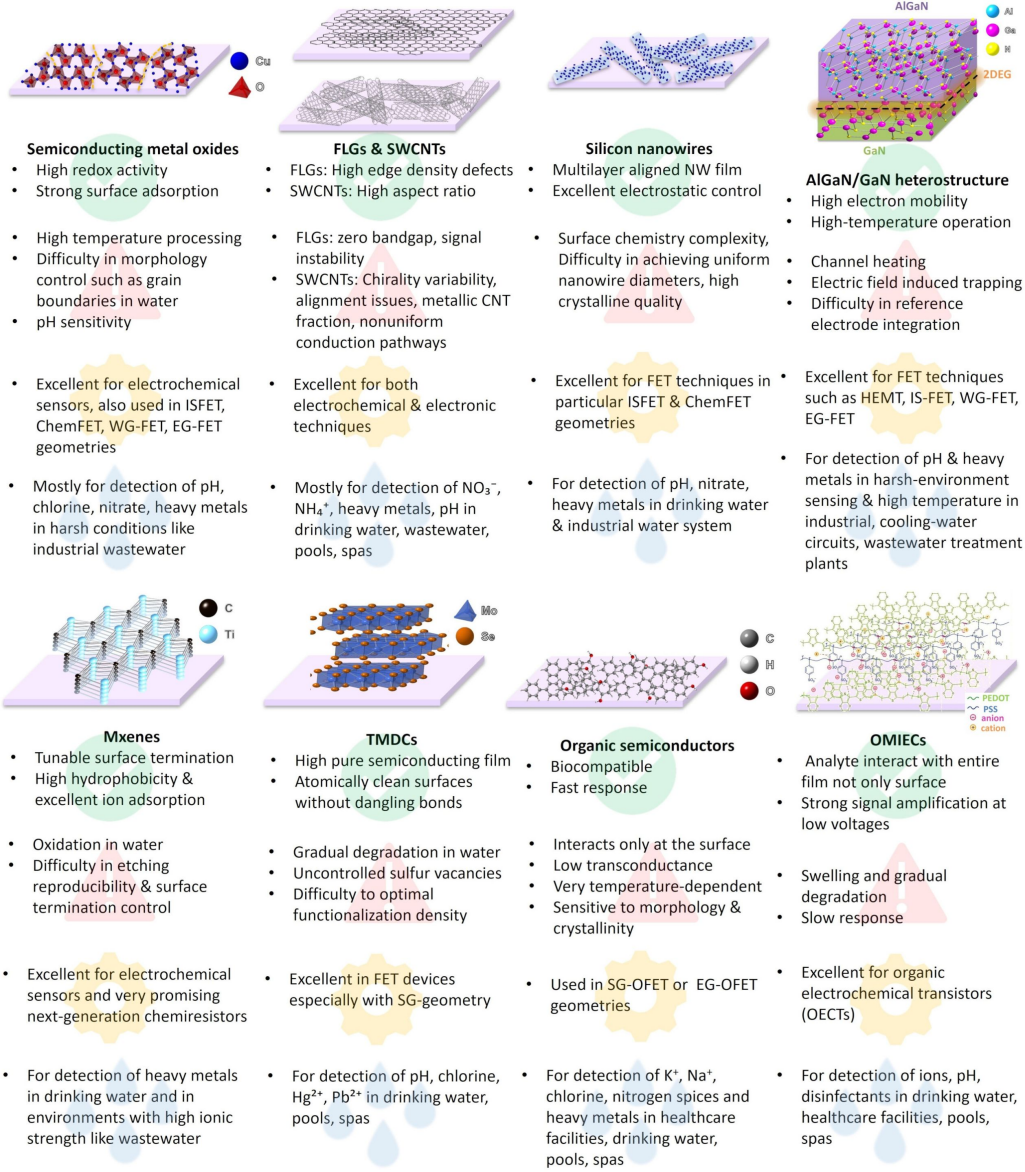
Summary of the properties of classified active-layer materials, highlighting their representative structures, key advantages, challenges specific to aqueous environments, compatible device types and geometries, and demonstrated applications in ion sensing. Images of material structures were generated using CrystalMaker^®^: a crystal and molecular structures program for Mac and Windows. CrystalMaker Software Ltd, Oxford, England (https://www.crystalmaker.com). This content is not subject to CC BY 4.0.

Table 3 provides a concise comparison of the electronic properties and specific surface areas of the active material classes reviewed in this work. Figure 12 complements this by summarizing each material category through representative structures or mesoscopic film morphologies, their key advantages and dominant challenges in aqueous media, the device geometries they are most compatible with, and their demonstrated sensing applications. Table 4 further evaluates the performance of sensors fabricated with these materials, including sensitivity, selectivity, water stability, response time, scalability, and fabrication cost. Overall, most active materials used for electrochemical and electronic ion detection are inorganic, owing to their intrinsically higher conductivity, charge mobility, and surface area relative to their organic counterparts (Table 3). As shown in Table 4, organic thin films generally exhibit lower sensitivity, selectivity, and long-term stability in water, despite their fast response time and low-cost fabrication, whereas OMIECs-based channels offer more practical performance for ion sensing. However, OMIECs in OECTs typically suffer from slower ion transport (leading to longer response times), potential issues with long-term chemical stability in harsh aqueous media, and often lower mobility compared to inorganic semiconductors. In contrast, inorganic channel materials deliver higher carrier mobilities, sharper gating behavior (higher on/off ratios), better chemical and thermal stability under aggressive conditions, and well-established microfabrication/integration through semiconductor processing. The trade-off for inorganic materials is often that ion-to-electronic coupling must rely on surface gating (rather than bulk volumetric coupling), which places constraints due to Debye screening in high-ionic-strength water; also, fabrication can be more complex and costlier when scaling to large-area or flexible platforms. In summary, organic channels offer excellent integration and low-voltage operation but face durability and speed challenges, while inorganic channels deliver high performance and robustness but may require more complex gating architectures and fabrication to function optimally in real-world aqueous ion sensing. Regarding stability, most inorganic semiconductors (except metal oxides and MXenes) maintain their properties in water while organic semiconductors, metal oxides and MXenes may gradually change due to hydration, oxidation, or ion intercalation, causing baseline drift. High-quality and well-passivated graphene, CNT, SiNW, MXene, TMDC FETs, and AlGaN/GaN HEMTs show low hysteresis, but amorphous metal oxides in IS-FET geometry, or conducting polymer channels in OECTs can show higher noise and hysteresis unless surface/interface engineering is applied. Between all introduced channel materials, AlGaN/GaN heterostructures and SiNWs are the most electrically robust and stable channels under continuous gating [105,173,227]. In conclusion, the choice of an appropriate sensing material depends on the intended detection mechanism, the practical constraints of the fabrication process, and the specific requirements of the target application. The advantages of both organic and inorganic materials can be combined by fabricating channels from hybrid materials for instance OMIEC–CNT composites. Using this strategy, mobility or electronic conduction via conductive fillers can be improved, ion uptake via polymer matrix can be enhanced, and the morphology can be tuned for balancing ion access vs. electronic conduction [173,228]. Devices can also be integrated with advanced materials such as COFs, MOFs, and IIPs to simultaneously achieve high conductivity and high selectivity. Their intrinsic properties are summarized in Table 3, and their sensing performance is evaluated in Table 4. Notably, IIPs meet many criteria of an ideal ion sensor, that is, high sensitivity, strong selectivity, excellent water stability, scalability, cost-effectiveness, and reproducibility, highlighting their potential for practical deployment.

**Table 3:** Comparison of the electronic properties and specific surface area of classified materials.

Material	Bandgap (eV)	Electrical conductivity (S/m)	Charge mobility (cm^2^/(V·s))	Specific surface area (m^2^/g)	Ref.

SMOs	3.0–4.0	10^−6^–10^2^	0.1–50	20–200 (nanostructured)	[229]
FLG	≈0 (semi-metal)	10^4^–10^5^	1000–50000 (high-quality)	300–800	[230]
SWCNTs	0.5–1.5	10^4^–10^6^	10000–100000	200–800	[20]
SiNWs	1.1–2.0 (bulk to strongly confined)	10^2^–10^4^	50–1500	10–100	[28]
AlGaN/GaN heterostructures	—	10^4^–10^5^ (2DEG sheet conductivity)	1000–2000	—	[142]
MXenes	0–0.3	10^4^–10^5^	1–100	50–300	[154]
TMDCs	1.6–2.1	10^−3^–10^2^	0.1–500	10–200	[159]
organic semiconductors	1.5–3.0	10^−8^–10^−3^	10^−3^–10	1–20	[170]
OMIECs	1.3–2.5	1–100 (doped)	10^−3^–1	not typically reported	[173]
COFs	1.5–3.0	10^−8^–1 (increases if doped)	0.1–10 (reported for some 2D COFs)	1000–3000	[202]
MOFs	2–5	10^−12^–1 (increases if doped)	very low–few	1000–7000	[198]
IIPs	3–5	10^−10^–10^−6^	very low	10–200	[205]

**Table 4:** Performance of classified materials for ion sensing in water, accompanied by an analysis of their device-fabrication considerations.

Material	Sensitivity	Selectivity	Water stability	Response time	Scalability	Fabrication cost	Ref.

SMOs	high	low (improves with surface ligands or composites)	high	moderate to fast	high	low (sol–gel, hydrothermal, screen-printing)	[229]
FLGs	high	low (improves with surface modification)	moderate (GO more hydrophilic, rGO more stable)	fast	high	moderate–high (CVD, transfer, functionalization)	[230]
SWCNTs	high	low (improves with surface modification)	high	fast	high	moderate (purification, dispersion, functionalization)	[20]
SiNWs	high	high with appropriate surface chemistry	high (native oxide stable in water)	fast	moderate to high (CMOS-compatible but cleanroom-intensive)	high (lithography, doping, passivation)	[28]
AlGaN/GaN	high	moderate to high with ISMs or receptors	high (chemically robust wide-bandgap surface)	fast	high (III–V wafer tech)	high (epitaxy, device processing)	[142]
MXenes	high	high	low (oxidation and structural degradation in water if not protected)	fast	high	moderate (etching, delamination, stabilization)	[154]
TMDCs	moderate to high	high if functionalized	moderate (surface oxidation/defects in aqueous media)	moderate to fast	moderate to high	moderate to high (CVD, exfoliation, printing)	[159]
organic thin films	moderate	moderate to high with specific ligands	low–moderate (swelling, fouling, degradation)	fast	high	low	[170]
OMIECs	high	moderate to high (depends on receptor design)	high if built on robust substrates and encapsulation	slow to moderate (often diffusion-limited through layers)	high	moderate (molecular design and device integration)	[173]
COFs	high	high	low to moderate (many COFs hydrolyze; some are water-stable)	moderate	moderate	moderate to high (synthesis and crystallinity control)	[202]
MOFs	high	high	low (many MOFs unstable in water; some water-stable variants exist	slow to moderate (diffusion into pores)	moderate	moderate	[198]
IIPs	high	high	high (cross-linked polymer networks stable in water)	moderate (binding and diffusion-limited)	high	low	[205]

Operating continuously in aqueous environments poses distinctive challenges. Biofouling of membranes, FET gates, channel materials, and chemiresistive films can impede mass transport, change surface charge, or add parasitic potentials, resulting in drift, hysteresis, and sluggish responses. The use of antifouling polymer coatings or flow-through cells, or the application of self-cleaning strategies can mitigate fouling-induced degradation [16]. For instance, robust solid-contact ISEs designed with antifouling layers and advanced transduction have improved stability for chloride, nitrate, ammonium, and heavy-metal monitoring in natural waters and treatment streams [12,16]. Another issue is that variations in the supporting electrolyte can change the solution ionic strength, alter the analyte activity coefficient, and may introduce competing ionic species to the system that interact with the sensor. To mitigate these effects, matrix-matched calibration or algorithm-based compensation strategies are required such as potentially leveraging ISFET arrays and machine-learning models [8,10]. Moreover, variations in temperature and flow rate change both thermodynamics and kinetics, perturbing the ion transport and reference electrode stability. Using multiparameter sensing and algorithmic compensation can help to stabilize outputs [17]. Flow-through ISFET systems for automated ammonium monitoring and high-transconductance nitrate ISFETs further highlight progress toward deployments that couple microfabrication with rugged fluidic packaging [11,231]. Furthermore, the requirement for stable reference electrodes remains a critical challenge in continuous sensing platforms, particularly when miniaturized. In response, significant research is directed toward solid-state reference technologies and reference-free or self-calibrating sensing methodologies [7,13,15]. As an example, ion-selective-membrane-coated chemiresistive devices have achieved LODs around 1–2 µg/L for Pb^2+^ in water during continuous operation, indicating a viable path for low-cost monitoring of trace concentration of heavy metals [103]. In addition, many sensing materials suffer from significant contact-resistance issues at the terminals, and/or require proper passivation layers to remain stable. These factors demand careful materials selection and precise engineering strategies to ensure reliable device performance. Last, long-term drift from material aging, membrane leaching, or interfacial redox phenomena remains a major barrier, making self-diagnostics and autonomous recalibration highly valuable in multiweek monitoring [14–15,17].

## Conclusion

This review highlights the diverse landscape of materials and strategies employed in solid-state ion sensing, emphasizing both their potential and limitations. As the field advances, it is increasingly clear that no single material system will dominate all sensing scenarios. Instead, progress will depend on smart choices of channel material, interfacial chemistry, and sensing architecture, which is dictated by the target analyte, operating environment, and required performance metrics such as sensitivity, selectivity, stability, and response time. Metal oxides, while not commonly used as FET channel materials due to their wide bandgap and abundance of surface states, remain valuable as redox-active components in nanocomposites that enhance electrochemical signal transduction. Their catalytic activity and chemical robustness make them particularly well suited for oxidizing or reducing analytes such as heavy-metal ions, reactive oxygen species, and disinfectants. Carbon-based materials such as graphene and carbon nanotubes and their functionalized derivatives offer high sensitivity owing to their high transconductance and strong surface-charge coupling. These materials are well suited for detecting low-concentration analytes such as trace metal ions or biologically relevant ions in buffered systems. However, future efforts must prioritize improved control over synthesis, surface chemistry, and device fabrication to overcome reproducibility challenges that currently limit large-scale deployment and commercialization. In contrast, silicon nanowires and AlGaN/GaN channels demonstrate excellent stability. For applications requiring long-term stability and minimal drift, such as continuous water quality monitoring or implantable and wearable biosensors, semiconductor platforms like silicon nanowires and AlGaN/GaN heterostructures are likely to remain dominant. Their inherent robustness, mature fabrication processes, and compatibility with existing electronics infrastructure make them particularly appealing for commercialization and field deployment although their device-fabrication workflows remain relatively complex and costly. MXenes provide high electrical conductivity and high surface activity, making them attractive for rapid and highly sensitive ion detection. However, they suffer from instability in aqueous environments due to surface terminations. Future research must focus on surface passivation strategies, compositional tuning, or hybridization with stabilizing matrices to unlock their full potential for real-world sensing applications. TMDCs stand out as a versatile class of semiconductors whose tunable bandgap and atomically thin structure, enable stable and sensitive electrical sensing platforms, most commonly implemented in SG-FET geometries.

Conventional organic FETs, based on small molecules or conjugated polymers, generally exhibit lower sensitivity compared to inorganic materials, which limits their use in ultratrace detection. However, they offer a number of advantages such as rapid response times, mechanical flexibility and biocompatibility, making them attractive for transient sensing, wearable platforms, and low-cost disposable devices. OMIECs attempt to overcome the weaker performance of conventional OFETs by incorporating volumetric electrochemical interactions, thereby significantly enhancing sensitivity. This improvement in sensitivity, however, comes at the expense of response speed, which highlights a broader challenge in OMIECs, that is, the need to decouple ion transport kinetics from electronic response. Future material design efforts should aim to optimize polymer morphology, ion mobility, and device architecture to achieve both high sensitivity and rapid temporal resolution. Looking ahead, application-specific material selection will become increasingly important. For example, environmental monitoring of pH, heavy metals, and nutrients benefits from stable inorganic channels (SiNWs, GaN) or robust carbon-based materials functionalized with chelating ligands. Biomedical sensing of electrolytes is well served by OMIECs, TMDCs, and CNT-based FETs functionalized with biomolecular receptors. Industrial process control, which often involves harsh chemical environments, may rely on metal-oxide nanocomposites or protected MXene-based architectures. Wearable and flexible sensing aligns naturally with organic semiconductors, and hybrid polymer–nanomaterial composites.

Beyond channel materials, surface engineering plays a critical role in optimizing sensor performance. The deliberate introduction of defects and nanoparticle dopants enhances ion diffusion, charge transfer, and signal amplification. At the same time, covalent or noncovalent functionalization with organic molecules can improve operational stability and selectivity. However, lack of tight control over interfacial structure and defects remains a major source of noise, hysteresis, and drift. A deeper mechanistic understanding of ion–interface interactions, supported by in situ characterization and modeling, will be essential to rationally designing stable and low-noise sensor interfaces. Nanocomposites offer an alternative to surface functionalization, with the potential to either mitigate or exacerbate interface issues depending on their phase distribution, percolation pathways, and interfacial compatibility. As such, future work should focus on predictive design rules that link nanocomposite structure to sensing performance.

Advanced porous materials including MOFs, COFs, and IIPs are expected to play an increasingly important role in achieving high selectivity. Their well-defined pore structures, large surface areas, tunable chemistry, and predictable sensing mechanisms make them particularly attractive for discriminating among chemically similar ions. Given their intrinsically low electrical conductivity, these materials are best deployed as functional layers or composite components integrated with conductive or semiconductive channels. Biomolecular recognition elements deliver excellent selectivity. However, their limited operational lifetime, susceptibility to denaturation, and high cost constrain their widespread adoption. Future directions may include the development of biomimetic or synthetic analogues that retain selectivity while improving robustness and shelf life. Ion-selective membranes remain one of the most effective strategies for achieving stable and selective ion sensing, particularly in long-term or fouling-prone environments. They are necessary when film protection and ion buffering are required such as in physiological matrices or wastewater monitoring. However, in applications where direct channel–analyte interactions are essential for sensitivity or response speed, membrane integration may be unnecessary or even detrimental. A more detailed understanding of when and how to deploy membranes will be critical for optimal sensor design.

At the system level, sensor arrays represent a powerful approach for multiplexed detection and signal amplification. Arrays of single-analyte sensors are powerful when absolute accuracy and direct readouts are required, such as in clinical diagnostics or regulatory water monitoring. Conversely, cross-reactive arrays are more flexible for complex mixtures where single-analyte selectivity is neither feasible nor necessary, though they rely on computational models such as machine learning, chemometric analysis, and pattern-recognition algorithms for interpretation. In summary, the future of solid-state ion sensing lies in the convergence of tailored material selection, sophisticated interface engineering, and intelligent system integration. Continued progress will require interdisciplinary collaboration across chemistry, material engineering, electrical engineering, and data science. By embracing application-specific design principles and leveraging the complementary strengths of diverse material classes, the field is well positioned to deliver robust, real-time, and scalable ion sensors capable of operating in complex aqueous environments.

## Data Availability

Data sharing is not applicable as no new data was generated or analyzed in this study.
